# Ammonia Borane All-In-One Modification Strategy Enables High-Performance Perovskite Solar Cells

**DOI:** 10.1007/s40820-025-01951-6

**Published:** 2026-01-02

**Authors:** Jiaxin Ma, Cong Shao, Yirong Wang, Guosheng Niu, Kaiyi Yang, Yao Zhao, Fuyi Wang, Zongxiu Nie, Jizheng Wang

**Affiliations:** 1 Beijing National Laboratory for Molecular Sciences CAS Key Laboratory of Organic Solids, Institute of Chemistry, Chinese Academy of Sciences, Beijing, 100190 People’s Republic of China; 2 School of Chemical Sciences, University of Chinese Academy of Sciences, Beijing, 100049 People’s Republic of China; 3 CAS Key Laboratory of Engineering Plastics, Institute of Chemistry, Chinese Academy of Sciences, Beijing, People’s Republic of China; 4 Beijing National Laboratory for Molecular Sciences, National Centre for Mass Spectrometry in Beijing, CAS Key Laboratory of Analytical Chemistry for Living Biosystems, Chinese Academy of Sciences, Beijing, People’s Republic of China

**Keywords:** Ammonia borane, All-in-one modification, Multifunctional complex, Perovskite solar cells

## Abstract

Perovskite solar cells have achieved remarkable progress in photovoltaic efficiency. However, interfacial defects at the buried and upper interfaces of perovskite layer remain a critical challenge, leading to charge recombination, ion migration, and iodine oxidation. To address this, we propose a novel all-in-one modification strategy employing ammonia borane (BNH_6_) as a multifunctional complex. By incorporating BNH_6_ at both buried and upper interfaces simultaneously, we achieve dual-interfacial defect passivation and iodide oxidation suppression through three key mechanisms: (1) hydrolysis-induced interaction with SnO_2_, (2) coordination with Pb^2+^, and (3) inhibition of I^−^ oxidation. This approach significantly enhances device performance, yielding a champion power conversion efficiency (PCE) of 26.43% (certified 25.98%). Furthermore, the unencapsulated device demonstrates prominent enhanced operation stability, maintaining 90% of its initial PCE after 500 h under continuous illumination. Notably, our strategy eliminates the need for separate interface treatments, streamlining fabrication and offering a scalable route toward high-performance perovskite photovoltaics.

**Supplementary Information:**

The online version contains supplementary material available at 10.1007/s40820-025-01951-6.

## Highlights

- An all-in-one modification strategy was developed by introducing a multifunctional complex ammonia borane (BNH_6_) into the buried and upper interfaces simultaneously.
- BNH_6_ uniquely realizes dual-interfacial defect passivation and iodide oxidation suppression by interacting with SnO_2_ through hydrolysis, coordinating with Pb^2+^ and inhibiting the oxidation of I^−^.
- The optimized perovskite solar cells achieve a champion efficiency of 26.43% (certified, 25.98%) with negligible current density–voltage hysteresis and significantly improved thermal and light stability.

**Supplementary Information:**

The online version contains supplementary material available at 10.1007/s40820-025-01951-6.

## Graphical abstract

**Figure:**
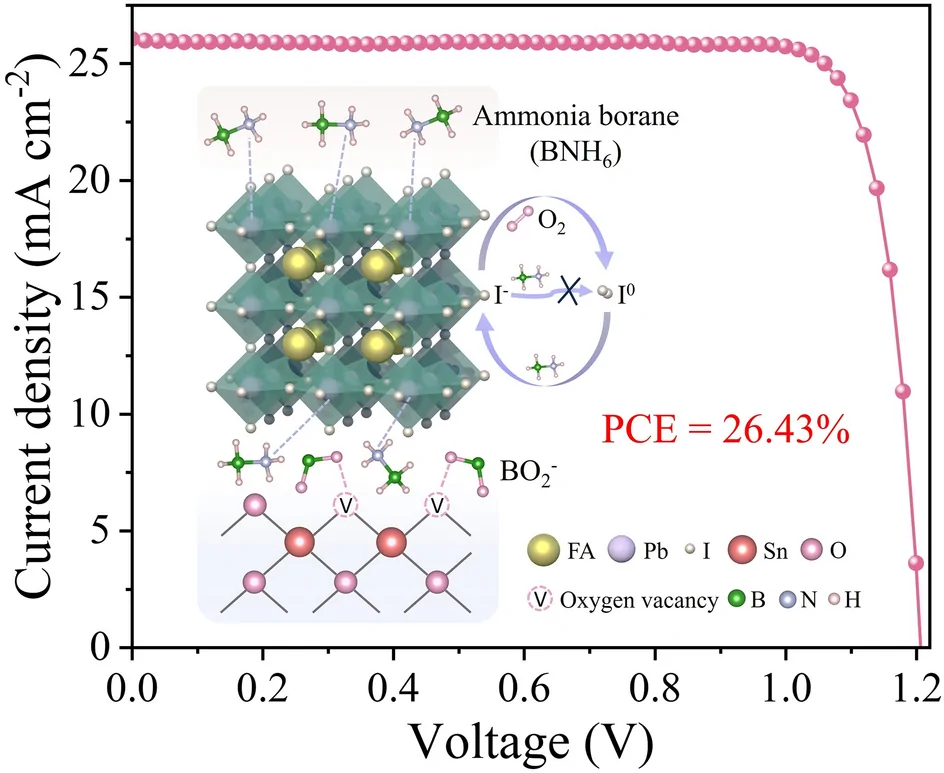

## Introduction

Organic–inorganic hybrid perovskite solar cells (PSCs) have become one of the most attractive fields due to their excellent optoelectronic properties [1–5]. The power conversion efficiency (PCE) of PSCs has rapidly reached 27.0% in the past few years [6–8]. This continuous progress is attributed to various attempts, including bandgap modulation [9–11], crystallization behavior regulation [12–17], and interface modification [18–27]. The low-temperature solution method is prevalent for preparing state-of-the-art PSCs. However, perovskite films prepared through such rapid low-temperature approach are liable to generate large numbers of interfacial defects, which serve as nonradiative recombination centers to hinder charge transport [28–31]. To address this, various strategies have been explored. For example, Yi et al. employed 1-[3-(Trimethoxysilyl)propyl]urea (TMPU) at SnO_2_/perovskite interface and trimethoxy (3,3,3-trifluoropropyl)silane (TMFS) at perovskite/Spiro-OMeTAD interface to passivate detrimental interface defects and facilitate faster carrier extraction [32]. Zheng et al. reported a strategy to dense the hole transport layer (HTL) by introducing (aminomethyl)phosphonic acid (AMP) into the precursor solution, concurrently modifying the top surface of the perovskite with 2-(3-fluorophenyl)ethylamine iodide (mF-PEAI) and piperazinium diiodide (PDI) [33]. These effective interface modification strategies are summarized in Table S1. However, the applying of excessive types of additives is unfavorable to the rapid device manufacturing, as well as the commercial fabrication.

Besides, iodide ions (I^−^) in the perovskite layer can migrate under the influence of environmental factors due to their lower formation energy [34]. This migration can lead to an uneven ion distribution at the interfaces, and the accumulation of iodine ions at the interfaces may induce adverse redox reactions, which frequently involve the generation of iodine defects (I^0^) [35–37]. I^0^ is volatile and can easily escape from the perovskite layer, accelerating the degradation of perovskite. Nevertheless, only a few additives were used to address this issue, such as benzylhydrazine hydrochloride (BHC) [38], the redox pairs of Eu^3+^-Eu^2+^ [35], and fluoroN,N,N″,N″-tetramethylformamidinium hexafluorophosphate (TFFH) [39], serving as reducing agents effectively reduced I^0^ back to I^−^. In spite of these attempts have been made in improving the performance of PSCs, these works are all achieved by improving the stability of perovskite precursor solutions. However, when perovskite film is exposed to ambient environment, I^−^ at the interface is more likely to be oxidized and result in defects, making the elimination of interface defects still challenging.

**Fig. 1:**
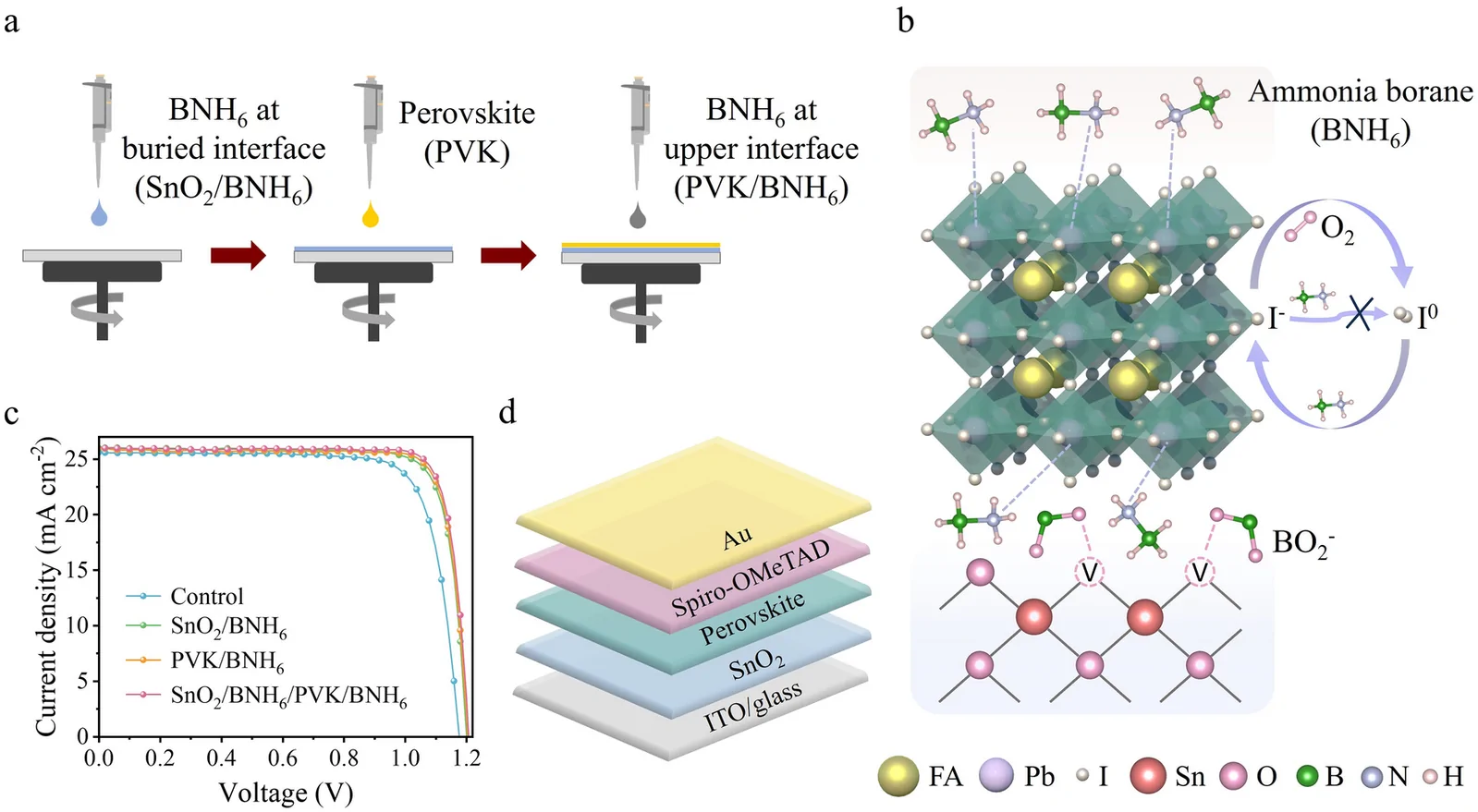
Structure and preparation of BNH_6_ in perovskites. **a** Schematic of device fabrication processes. SnO_2_/BNH_6_ and PVK/BNH_6_ are defined as BNH_6_ at the buried and upper interfaces of perovskite layer, respectively. **b** Mechanism diagram of BNH_6_ modification at different positions. **c** *J* − *V* curves of PSCs under various BNH_6_ treatment conditions. **d** Device structure diagram of a typical *n-i-p* PSC

In this work, an all-in-one modification strategy was developed to address these issues. This strategy is achieved by incorporating ammonia borane (BNH_6_) into the buried (SnO_2_/BNH_6_) and upper (PVK/BNH_6_) interfaces of perovskite layer, respectively (Fig. 1a) to realize dual-interfacial defect passivation and iodide oxidation suppression. BNH_6_ is a unique molecular complex composed of electron-rich nitrogen elements and electron-poor boron elements that determine its multifunctionality. BNH_6_ enables the interaction with SnO_2_ through hydrolysis, passivates the uncoordinated Pb^2+^ to realize dual-interfacial optimization, and acts as a reducing agent to reduce I^0^ back to I^−^ to inhibit iodide oxidation (Fig. 1b). These unique properties enable BNH_6_ to play an effective role under different treatment conditions (Fig. 1c). Consequently, a typical *n-i-p* PSC (Fig. 1d) with BNH_6_ all-in-one modification achieved a champion efficiency of 26.43% (certified, 25.98%) with negligible current density − voltage (*J* − *V*) hysteresis. Furthermore, the unencapsulated device demonstrates prominent enhanced operation stability, maintaining 90% of its initial PCE after 500 h under continuous illumination. This work presents a simple and highly effective strategy to address dual-interface defects in perovskite films and eliminate the complexity of multi-additive systems, offering a promising pathway to enhance the compatibility with rapid manufacturing and commercialization.

## Experimental Section

### Materials

All the chemicals were purchased from commercial vendors without further purification. SnO_2_ colloid precursor (tin (IV) oxide, 15% in H_2_O), N,N-dimethylformamide (DMF, 99.8%) and dimethyl sulfoxide (DMSO, 99.8%) were purchased from Alfa Aesar. Chlorobenzene (99.8%) and isopropanol (99.5%) were purchased from Sigma-Aldrich. Acetonitrile (99.9%) was purchased from Acros. CsI (99.999%) was purchased from Sigma-Aldrich. PbI_2_ (99.999%), FAI (99.9%) and MACl (99.9%) were purchased from Advanced Election Technology Co., Ltd. MeO-PEAI (99%), Spiro-OMeTAD (99.9%), 4-tert-butylpyridine (96%) and LiTFSI (99%) were purchased from Xi’an Polymer Light Technology. Ammonia borane was purchased from Aladdin Bio-Chem Technology Co., Ltd.

### Solution Preparation

Tin (IV) oxide colloid solution (15 wt%) was diluted with deionized water (volume ratio: 1:2). The BNH_6_ solution (1 mg mL^−1^) coated at the buried interface of perovskite was prepared by dissolving 1.0 mg BNH_6_ in 1 mL deionized water. For the Cs_0.05_FA_0.95_PbI_3_ precursor solution, 1.4 M FAI, 0.07 M CsI, 1.58 M PbI_2_ and 0.49 M MACl were mixed in the solvent of DMF and DMSO (volume ratio: 8:1) and stirred at room temperature for 6 h. The BNH_6_ solution (1 mg mL^−1^) coated at the upper interface of perovskite was prepared by dissolving 1.0 mg BNH_6_ in 1 mL IPA. The MeO-PEAI solution (3 mg mL^−1^) was prepared by dissolving 3 mg MeO-PEAI in 1 mL IPA. The Spiro-OMeTAD solution was prepared by dissolving 72.3 mg of Spiro-OMeTAD, 28.8 μL of t-BP, and 35 μL of Li-TFSI solution (260 mg mL^−1^, in acetonitrile) in 1 mL chlorobenzene.

### Device Fabrication

The ITO substrate was cleaned in deionized water, acetone, and ethanol in sequence, followed by being treated with oxygen plasma for 10 min. Next, the SnO_2_ colloid solution was spin-coated onto the substrate at 4000 rpm for 30 s and annealed at 150 °C for 30 min in ambient to form the SnO_2_ film. Then the BNH_6_ solution (dissolved in deionized water) was spin-coated on the SnO_2_ film at 4,000 rpm for 30 s and annealed at 150 °C for 10 min in ambient to form the SnO_2_/BNH_6_ film. After cooling down, the SnO_2_ or SnO_2_/BNH_6_ films were treated with oxygen plasma (60 W) for 5 min. Then the perovskite solution (60 μL) was spin-coated at 1000 rpm for 10 s and 5000 rpm for 30 s. At 20 s from the last, 700 μL diethyl ether (DEE) as an antisolvent was rapidly dropped onto the substrate. Then the perovskite precursor film was annealed at 120 °C for 1 h under an ambient atmosphere with ~ 25% RH. After the perovskite film was cooled down, the sample was transferred to a nitrogen-filled glove box for further processing. The BNH_6_ solution (dissolved in IPA) was spin-coated on the perovskite film at 4000 rpm for 30 s. Then the MeO-PEAI solution (100 μL) was spin-coated for passivation at 4000 rpm for 30 s. Then the perovskite film was annealed at 100 °C for 5 min. The Spiro-OMeTAD solution (50 μL) was spin-coated onto the perovskite layer at 4000 rpm for 30 s. Finally, 80 nm Au electrode was deposited by thermal evaporation.

### Density Functional Theory Calculation

Theoretical calculations were performed with the Vienna ab initio simulation package (VASP). The exchange–correlation energy is described by the Perdew-Burke-Ernzerhof (PBE) form of generalized-gradient approximation (GGA) exchange–correlation energy functional. The structure optimizations of systems of BH_3_NH_3_ before and after adsorption on PbI_2_ and FAI terminal FAPbI_3_ (100) surfaces have been carried out by allowing top layer atomic positions to vary and fixing lattice parameters and bottom layer atomic positions until the energy difference of successive atom configurations was less than 10^−6^ eV. The force on each atom in the relaxed structures was less than 0.015 eV Å^−1^. The cutoff energy for the plane-wave basis set was set to 400 eV. The k-point spacing was set to be smaller than 0.03 Å^−1^ over Brillouin zone (BZ).

### Measurements

The current density − voltage (*J* − *V*) characteristics of the devices were measured using a Keithley 2420 under AM 1.5 sunlight at an irradiance of 100 mW cm^−2^ provided by a solar simulator (Newport, Oriel Sol3A Class AAA, 94043A). Light intensity was calibrated using a monocrystalline silicon reference cell with a KG5 window (Newport, Oriel 91,150). The J − V curves were obtained with a scan rate of 100 mV s^−1^ and a scan step of 20 mV from 1.22 to 0.00 V (reverse) or from 0.00 to 1.22 V (forward). The area of the cell is 0.1225 cm^2^ and a mask of 0.09881 cm^2^ (certificated by NIM, China. The certificate No.: CDjc2023 − 08390) was used to determine the effective area of the device before the test. Heating acceleration: Unencapsulated devices were heated at 65 ± 3 °C in a nitrogen atmosphere (ISOS-T-1). Long-term light stability tests: Unencapsulated devices were treated by white light-emitting diode (LED) with an intensity of 100 mW cm^−2^ at 23 ± 3 °C in a nitrogen atmosphere (ISOS-L-1). EQE measurements were recorded by an Enli Technology EQE system, which was calibrated by a certified silicon solar cell. The scan interval was 5 nm, and there was no bias light and mask used during the measurement. The top-view and cross-sectional SEM images were obtained using Hitachi S-4800 at the accelerating voltage of 5.0 kV. The surface roughness of perovskite film was measured by an AFM (Nanoscope V, Vecco) in tapping mode under the ambient atmosphere. The XRD patterns were taken on PANalytical Empyrean with a Cu Kα radiation (*λ* = 1.5406 Å) and a scanning rate of 5° min^−1^ in the 2θ range of 5°–50° at a step size of 0.02 s. 2D-XRD spectra were measured using a Rigaku SmartLab X-ray diffractometer with Cu Kα1 (1.54060 Å) and a HyPix-3000 2D hybrid pixel array detector. All samples for XRD testing were prepared on quartz glass substrates. The steady PL spectra was recorded by Horiba FluoroMax + fluorescence spectrometer with an excitation at 490 nm. Time-resolved PL was carried out by the FLS980 fluorescence spectrometer with excitation wavelength at 485 nm. ToF–SIMS profiling measured the depth distributions of the negative ions with perovskite on the ITO substrate. The samples were analyzed using a ToF–SIMS 5 instrument (IONTOF) with a Bi + primary beam (10 keV and 1 pA) and Cs + sputter beam (3 keV and 5 nA). The sputter size was 100 μm × 100 μm. The UV absorption spectra of perovskite films and FAI solutions were measured using a Hitachi U3900 spectrophotometer. XPS/UPS measurements were obtained using an XPS/UPS system (ESCALAB250XI, Thermo Fisher Scientific). LEIPS measurement was performed on a customized ULVAC-PHI LEIPS instrument with Bremsstrahlung isochromatic mode. FTIR were performed on a HITACHI F-4500IR spectrometer with samples prepared as KBr tablets. The mass ratio of the sample to KBr is 1:100. Raman spectra were recorded using an NT-MDT NTEGRA Spectra system. EIS and M-S tests were measured with an electrochemical workstation (Modulab XM, USA). ^1^H NMR spectra were recorded on a Bruker Avance 400 Spectrometer. The concentration of the sample is 10 mg mL^−1^ in DMSO-*d*_*6*_.

### Calculation Methods

#### Conductivity and Electron Mobility

The conductivity (σ) was calculated using Eq. (1):

1 $$\begin{array}{*{20}c} {\sigma = \frac{I}{V}} \\ \end{array}$$

where I is the current, V is the voltage.

The electron mobility ($\mu_{e}$) was calculated using Eq. (2):

2 $$\begin{array}{*{20}c} {J = \frac{9}{8}\varepsilon \varepsilon_{0} \mu \frac{{V^{2} }}{{L^{3} }}} \\ \end{array}$$

where J is the current density, ε is the relative dielectric constant, $\varepsilon_{0}$ is vacuum permittivity, V is the voltage, and L is the film thickness.

#### Residual Stress

The residual stress (σ) was calculated using Eq. (3):

3 $$\begin{array}{*{20}c} {\sigma = - \frac{E}{{2\left( {1 + v} \right)}}\frac{\pi }{180^\circ }\cot \theta_{0} \frac{{\partial \left( {2\theta } \right)}}{{\partial \left( {\sin^{2} \psi } \right)}}} \\ \end{array}$$

where E is the perovskite modulus (10 GPa), and v is the Poisson’s ratio of perovskite (0.3).

#### Ideality Factor

The diode ideality factor (n) was determined by fitting $V_{oc}$ as a function of light intensity using Eq. (4):

4 $$\begin{array}{*{20}c} {V_{{{\mathrm{oc}}}} = \frac{{nkT\ln \left( {I_{{{\mathrm{light}}}} } \right)}}{q} + C} \\ \end{array}$$

where n represents the ideality factor to single-molecule recombination, $I_{{{\mathrm{light}}}}$ is the light intensity, k is Boltzmann’s constant, T is temperature, and q is the elementary charge.

## Results and Discussion

### Interaction Between BNH_6_ and SnO_2_

**Fig. 2:**
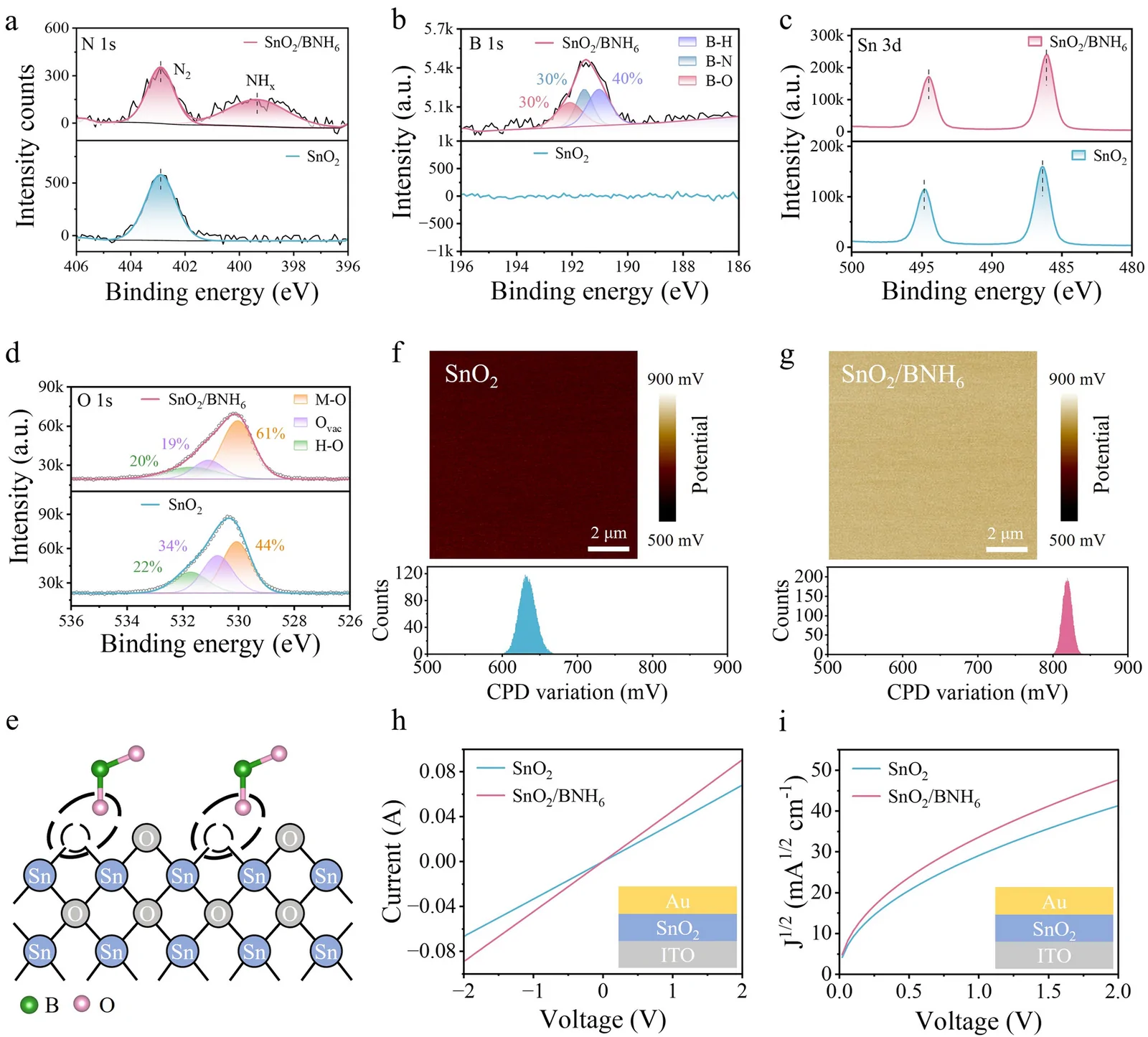
Interaction between BNH_6_ and SnO_2_. **a** N 1*s*, **b** B 1*s*, **c** Sn 3*d*, **d** O 1*s* XPS spectra of the SnO_2_ and SnO_2_/BNH_6_ films. **e** Schematic illustration of the occupation of BO_2_^−^ on O_V._ KPFM images of the **f** SnO_2_ film and **g** SnO_2_/BNH_6_ film. The statistical potential distributions of film surfaces are shown at the bottom. CPD, contact potential difference. **h** The electrical conductivity and **i** the electron mobility of the SnO_2_ and SnO_2_/BNH_6_ films. Inset shows the architecture of devices

The hydrolysis characteristic of BNH_6_ leads to the production of BO_2_^−^ (Fig. S1) [40], which can interact with SnO_2_. To further investigate the interaction between BNH_6_ and SnO_2_, X-ray photoelectron spectroscopy (XPS) was carried out for the SnO_2_ film (SnO_2_) and BNH_6_ treated SnO_2_ film (SnO_2_/BNH_6_). In the N 1*s* spectra (Fig. 2a), the peaks around 403 eV originate from molecular N_2_ adsorbed on the surface of SnO_2_, and the occurrence of NH_x_ around 399 eV in the SnO_2_/BNH_6_ film confirms that BNH_6_ was successfully introduced and hydrolyzed to produce NH_4_^+^ during annealing process. In the B 1*s* spectra (Fig. 2b), the signal of the SnO_2_/BNH_6_ film can be divided into three peaks. The peak around 192.1, 191.6, and 191.0 eV represents the B–O peak, B–N peak and B–H peak, respectively. To investigate the proportion of BO_2_^−^ produced by hydrolysis of BNH_6_, the value obtained by the integrated areas ratio of *S*_B−O_/(*S*_B−O_ + *S*_B−N_ + *S*_B−H_) is 30%, indicating that 30% of BNH_6_ molecules hydrolyze to produce BO_2_^−^. Besides, the above results indicate that most BNH_6_ molecules are not hydrolyzed and still exist in the form of B–N and B–H bonds, which will be beneficial for the subsequent film formation and defects passivation of perovskite. The Sn 3*d* peaks at 486.4 and 494.8 eV for the SnO_2_ film shift to 486.1 and 494.5 eV for the SnO_2_/BNH_6_ film, respectively, which indicates the electron density increased after BNH_6_ treated owing to the negative charge on BO_2_^−^ (Fig. 2c). As revealed by O 1*s* spectra (Fig. 2d), the signals of the SnO_2_ and SnO_2_/BNH_6_ films can be fitted into three peaks. The peak at about 530 eV is assigned to the lattice oxygen (M–O), representing the fully coordinated oxygen atoms within the SnO_2_ crystal lattice. The peak at about 531 eV is attributed to oxygen vacancies (O_vac_), which is associated with defect-related states. And the peak at about 532 eV corresponds to surface hydroxyl groups or adsorbed water [41, 42]. It is easily found that the relative intensities of M–O increased (from 44% to 61%), as well as O_vac_ (from 34% to 19%) and H–O (from 22% to 20%) decreased after BNH_6_ modification, indicating suppressed nonradiative recombination and enhanced electron mobility [43], which is attributed to the occupation of BO_2_^−^ on O_vac_ (Fig. 2e). In addition, during the actual device fabrication process, the O 1*s* XPS spectra of SnO_2_ and SnO_2_/BNH_6_ films treated by oxygen plasma exhibit higher M–O relative intensity and lower O_vac_ and H–O relative intensities compared to the untreated films (Fig. S2). Correspondingly, the performance of the resulting devices is also enhanced (Fig. S3 and Table S2). These features suggest a strong interaction between BNH_6_ and SnO_2_ due to the hydrolysis product of BNH_6_ (BO_2_^−^), interacting with the uncoordinated Sn^4+^ by filling O_V_ through adsorption. In addition, the SnO_2_ and SnO_2_/BNH_6_ films were subjected to X-ray diffraction (XRD) characterization. As shown in Fig. S4, the position and diffraction intensity of peaks are almost the same after BNH_6_ modification, illustrating that BNH_6_ does not damage the structure of the pristine SnO_2_.

To investigate the energy band structure of the SnO_2_ and SnO_2_/BNH_6_ films, ultraviolet photoelectron spectroscopy (UPS) and low energy inverse photoemission spectroscopy (LEIPS) were carried out. As shown in Fig. S5, the secondary electron cutoff edges (*E*_cutoff_) shifts from 16.72 to 16.82 eV after BNH_6_ modification, resulting in a decrease work function from 4.50 to 4.40 eV. Meanwhile, the conduction band (*E*_C_) shifts from − 4.44 to − 4.29 eV after the introduction of BNH_6_ (Fig. S6). The better matching of energy levels with perovskite for SnO_2_/BNH_6_ is expected to reduce interface energy barrier and thus suppress interface recombination. Then Kelvin probe force microscopy (KPFM) was measured to study the electrical charge distribution on the film surface, with contact potential difference (CPD) spectra shown in Fig. 2f, g. The SnO_2_/BNH_6_ film showed a higher CPD (819 mV) compared with the SnO_2_ film (634 mV), indicating a decrease in the work function and an increase of the Fermi level, which is consistent with the result of UPS.

In addition, the transmittance of SnO_2_ layer coated on indium doped tin oxide (ITO) glass is not affected with BNH_6_ modification (Fig. S7). And the absorption edge of SnO_2_ nearly does not change after BNH_6_ modificated (Fig. S8). The conductivity of the SnO_2_/BNH_6_ film (3.37 × 10^−3^ mS cm^−1^) is significantly higher than that of the SnO_2_ film (2.53 × 10^−3^ mS cm^−1^) (Fig. 2h), and the electron mobility of the SnO_2_/BNH_6_ film (3.18 × 10^−3^ cm^2^ V^−1^ S^−1^) is also greater than that of the SnO_2_ film (2.43 × 10^−3^ cm^2^ V^−1^ S^−1^) (Fig. 2i). The enhanced charge transport provides preferable electron diffusion and charge extraction, achieving higher open circuit voltage (*V*_OC_) and fill factor (FF) of PSCs. The atomic force microscope (AFM) measurement was carried out to study the root mean square (RMS), which is 1.21 and 0.80 nm for the SnO_2_ and SnO_2_/BNH_6_ films, respectively (Fig. S9). The reduced roughness of the SnO_2_/BNH_6_ film is attributed to the reduction in O_V_, which is conducive to facilitating optimal contact with perovskite film [44]. Additionally, the contact angle of the SnO_2_/BNH_6_ film (4.8°), is obviously smaller than that of the SnO_2_ film (7.2°), measuring by dropping perovskite precursor solution on the SnO_2_ and SnO_2_/BNH_6_ films, respectively (Fig. S10). The improved wettability indicates the better coverage for perovskite, which is consistent with the RMS results.

### Interaction Between BNH_6_ and FAI/PbI_2_

**Fig. 3:**
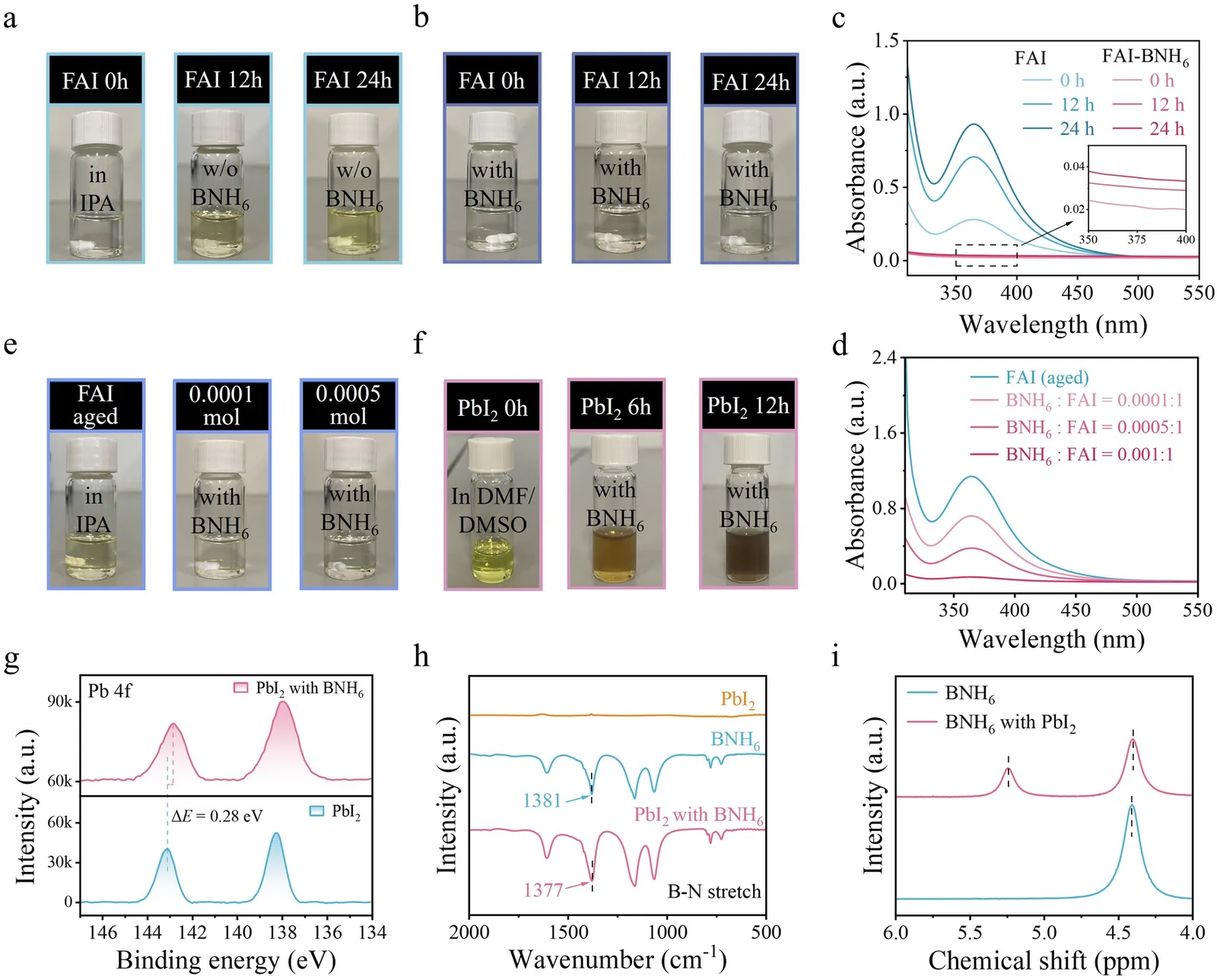
Interaction between BNH_6_ and FAI/PbI_2_. **a** The color change process of fresh FAI solution without BNH_6_ additive after ambient aging. **b** The color change process of fresh FAI solution with BNH_6_ additive after ambient aging. **c** UV–vis absorption spectra of fresh FAI solution with and without BNH_6_ after ambient aging. **d** UV–vis absorption spectra of aged FAI solution after introducing different concentrations of BNH_6_. **e** The color change process of aged FAI solution after introducing different concentrations of BNH_6_. **f** The color change process of PbI_2_ solution with BNH_6_ after ambient aging. **g** Pb 4*f* XPS spectra of PbI_2_ powders with and without BNH_6_. **h** FTIR spectra of PbI_2_, BNH_6_, and PbI_2_ with BNH_6_ films. **i** ^1^H NMR spectra of BNH_6_ and BNH_6_ with PbI_2_

The reaction between I^−^ and O_2_ has a thermodynamic tendency when the perovskite precursor solution is exposed to air, leading to the degradation of the precursor solution, thus seriously affecting the performance of devices [38]. Meanwhile, the depletion of I^−^ generates iodine interstitials accessibly, which is prone to form deep trapping states, causing more nonradiative recombination. To address this issue, BNH_6_ is applied and its function can be expressed by Fig. S11. We first attempted to mix BNH_6_ (0.001 mol) and FAI (1 mol) in 1 mL DMF to investigate the interaction between BNH_6_ and FAI. After continuous stirring along with 60 °C heating and air exposure, the transparent FAI solution without BNH_6_ turned to light yellow after 12 h aging and became bright yellow after 24 h aging (Fig. 3a). An absorption peak at 365 nm in the ultraviolet visible absorption spectroscopy (UV–vis) spectra gradually increased with aging time (Fig. 3c), which is ascribed to the oxidation of I^−^ into I^0^. Surprisingly, the color of FAI solution mixed with BNH_6_ remained transparent after 24 h aging (Fig. 3b), which is consistent with the result of almost no peak at 365 nm in the UV–vis spectra (Fig. 3c), demonstrating BNH_6_ can suppress the oxidation from I^−^ to I^0^. Importantly, yellow returned to transparent when BNH_6_ with increased concentrations were added to the aged FAI solution, accompanied by a notable reduction in the absorption peak observed at 365 nm (Fig. 3d, e). This result proves that the formed I^0^ can be reduced to I^−^ with the BNH_6_ addition. To further quantitatively demonstrate the effectiveness of BNH_6_ in inhibiting I^−^ oxidation, we have supplemented ion chromatography (IC) measurements. Specifically, we dissolved 8 mg of FAI in 100 mL of deionized water at ambient conditions and monitored the concentration of I^−^ at different aging times. As shown in Fig. S12, the I^−^ concentration gradually decreased with time, indicating progressive oxidation of I^−^ to I_2_. In contrast, after adding BNH_6_ to the solution, the I^−^ concentration remained almost unchanged over the same aging period, and was significantly higher than that in the control sample without BNH_6_. After 72 h, the I^−^ concentration in the sample without BNH_6_ decreased by 5.0%, while the I^−^ concentration in the sample with BNH_6_ addition decreased by only 0.7%. These results provide clear and quantitative evidence that BNH_6_ can effectively suppress the oxidation of I^−^ and thus mitigate the formation of elemental iodine. The reducibility of BNH_6_ is due to the H atom connected to the B atom can lose electrons and be oxidized to H_2_. The chemical reaction equation for BNH_6_ reducing I^0^ to I^−^ is speculated as follows:

5 $$\begin{array}{*{20}c} {2{\mathrm{NH}}_{3} \cdot {\mathrm{BH}}_{3} + 2{\mathrm{I}}^{0} \to 2{\mathrm{NH}}_{3} \cdot {\mathrm{BH}}_{2} {\mathrm{I}} + {\mathrm{H}}_{2} } \\ \end{array}$$

To explore the coordination between BNH_6_ and Pb–I framework, density functional theory (DFT) was employed to calculate the adsorption energies of BNH_6_ on the surface of FAPbI_3_ both with PbI_2_ and FAI terminations (Fig. S13). DFT calculations show that BNH_6_ is likely to bind to the FAI − terminated surface more stable with the adsorption energy of − 2.15 eV compared with the PbI_2_-terminated surface with the adsorption energy of − 1.03 eV.

Then BNH_6_ was added to PbI_2_ solution resulted in a noticeable color change after stirring for 6 h at room temperature. The color of the solution deepened significantly after 12 h (Fig. 3f) while the pristine PbI_2_ solution displayed no obvious alteration (Fig. S14), which is derived from the coordination between the N atom in BNH_6_ and Pb^2+^. To further testify this coordination, XPS, Fourier transform infrared spectroscopy (FTIR) and ^1^H nuclear magnetic resonance (NMR) were employed. In the XPS spectra (Fig. 3g), the Pb 4*f* peaks of PbI_2_ powders shift to lower binding energy after the introduction of BNH_6_, which is originated from the electron transfer from BNH_6_ to Pb^2+^, indicating the coordination of Pb–N. Additionally, FTIR analysis revealed shifts in the stretching vibration peaks of N–H (Fig. S15) and B–N (Fig. 3h) after mixing with PbI_2_, suggesting potential coordination interactions between BNH_6_ and Pb^2+^. Furthermore, in the ^1^H NMR spectra (Fig. 3i), the peak at 4.41 ppm, corresponding to the H atoms connected to B atoms in pure BNH_6_, splits into two peaks at 5.24 and 4.40 ppm upon the addition of PbI_2_. This shift suggests a strong interaction between BNH_6_ and PbI_2_, which alters the chemical environments of the H atoms in the BH_3_ group. This behavior is likely due to the breaking of B–N bonds in some BNH_6_ molecules allowing the N atom with lone pair electrons to coordinate with Pb^2+^ [45–47]. To quantify the extent of B–N bond breakage in BNH_6_ molecules during interaction with Pb^2+^ from the ^1^H NMR spectra, the following formula was applied:

6 $$\begin{array}{*{20}c} {{\mathrm{BNH}}_{6} \;{\mathrm{breakage}} \% = \frac{{S_{{{\mathrm{Pb}} - {\mathrm{N}}}} }}{{S_{{{\mathrm{Pb}} - {\mathrm{N}}}} + S_{{{\mathrm{B}} - {\mathrm{N}}}} }}} \\ \end{array}$$

where *S*_Pb−N_ represents the integrated area of the Pb–N peak, and *S*_B−N_ represents the integrated area of the B–N peak. The calculation result suggests that 29% of BNH_6_ molecules can additionally coordinate with Pb^2+^.

### Film Characterization

**Fig. 4:**
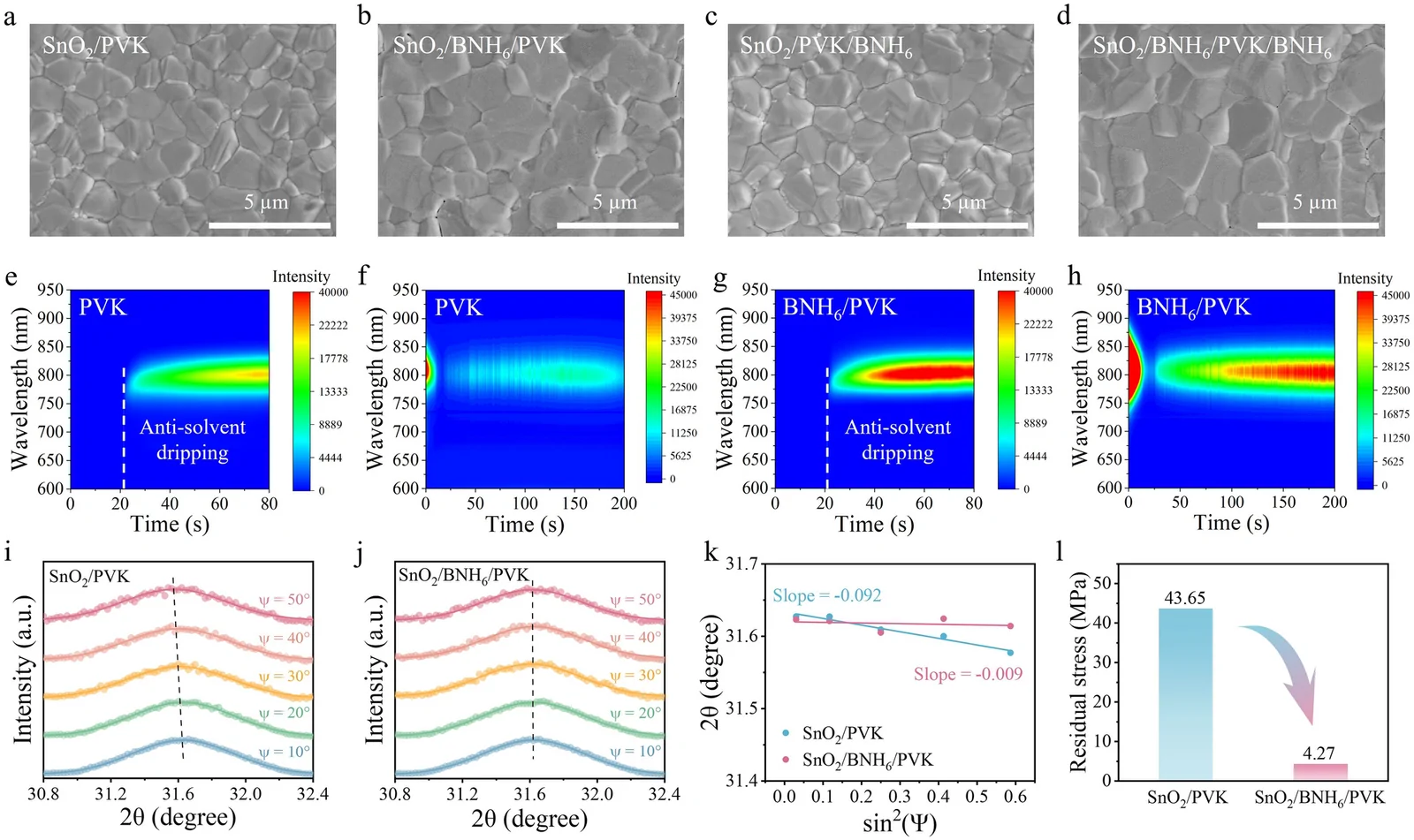
Characteristics of perovskite films. SEM images of **a** SnO_2_/PVK, **b** SnO_2_/BNH_6_/PVK, **c** SnO_2_/PVK/BNH_6_ and **d** SnO_2_/BNH_6_/PVK/BNH_6_ films. In situ PL spectra of the PVK film during the **e** spin-coating and **f** annealing process. In situ PL spectra of the BNH_6_/PVK film during the **g** spin-coating and **h** annealing process. GIXRD spectra at different Ψ angles (from 10° to 50°) of **i** SnO_2_/PVK and **j** SnO_2_/BNH_6_/PVK films. **k** Linear fit of 2θ–sin^2^ψ for the SnO_2_/PVK and SnO_2_/BNH_6_/PVK films. **l** Comparison of residual stress of the SnO_2_/PVK and SnO_2_/BNH_6_/PVK films

The morphology of perovskite films with and without BNH_6_ modification was investigated by scanning electron microscopy (SEM) and atomic force microscopy (AFM). SEM images (Fig. 4a–d) show that the perovskite film with BHN_6_ buried modification (SnO_2_/BNH_6_/PVK) exhibits significantly much larger grain size, indicating that BNH_6_ positively affects the crystallization of perovskite. While the grain size of the perovskite film with BNH_6_ upper modification (SnO_2_/PVK/BNH_6_) does not increase significantly. This is because BNH_6_ mainly plays the role of passivating Pb^2+^ and reducing I^0^ at the upper interface rather than regulating crystallization. The grain-size distributions of these films are displayed in Fig. S16. The pristine perovskite (SnO_2_/PVK) film has an average grain size of 1.59 µm, while the SnO_2_/BNH_6_/PVK, SnO_2_/PVK/BNH_6_ and the dual-interface modified (SnO_2_/BNH_6_/PVK/BNH_6_) films reveal larger size of 2.26, 1.70, and 2.15 µm, respectively. Meanwhile, the root mean square (RMS) roughness of SnO_2_/PVK film measured by AFM (Fig. S17) is 27.8 nm, and the reduced roughness in the SnO_2_/BNH_6_/PVK (23.5 nm), SnO_2_/PVK/BNH_6_ (24.7 nm) and SnO_2_/BNH_6_/PVK/BNH_6_ (19.0 nm) films is beneficial for defects suppression and charge extraction [47].

2D X-ray diffraction (2D-XRD) was conducted to explore the crystallization of perovskite film with and without BNH_6_ modification (Fig. S18). The results show that the (001) and (002) crystal planes of α-FAPbI_3_ are aligned parallel to the substrate normal, with no noticeable change across all the samples. Besides, the diffraction peaks of (001) and (002) crystal planes significantly enhanced for the SnO_2_/BNH_6_/PVK film, while no obvious changes are found for the SnO_2_/PVK/BNH_6_ film. The same phenomenon is observed from XRD patterns (Fig. S19). By comparison, the SnO_2_/BNH_6_/PVK/BNH_6_ film exhibits better crystal orientation of α-FAPbI_3_ for facilitating charge transfer. To further clarify the impact of BNH_6_ modification on the crystallization of perovskite, in situ photoluminescence (PL) measurement was performed during perovskite fabrication process. Figure 4e–h depicts the in situ PL spectra of perovskite films during spin coating and thermal annealing processes. During the spin coating stage, the BNH_6_/PVK film displays a stronger PL intensity in a shorter time upon anti-solvent dripping (Fig. 4e, g), implying the faster nucleation of α-FAPbI_3_ for the BNH_6_/PVK film [48, 49]. During the thermal annealing process (Fig. 4f, h), rapidly increasing PL intensities of the both films arise due to the generation of α-FAPbI_3_, and the subsequent sharp decrease can be mainly attributed to solvent volatilization and crystallization restructuring [50–52]. Then the re-enhanced and gradually stabilized PL intensity exhibits the dissolution–recrystallization equilibrium of α-FAPbI_3_ on the surface. The PL intensity of the BNH_6_/PVK film begins to sharply increase at 27 s, later than 19 s of the PVK film, indicating BNH_6_ effectively slows down the crystal growth. The rapid nucleation and delayed crystal growth of the BNH_6_/PVK film signifies higher quality perovskite with less nonradiative recombination, which is attributed to the ability of BNH_6_ to regulate the crystallization of perovskite.

The remarkable improvement in the crystallinity of the perovskite also affects the release of residual stress. Grazing-incidence X-ray diffraction (GIXRD) was investigated at various angles to assess the residual stress in perovskite films. The diffraction peaks of (012) crystal plane for the SnO_2_/PVK progressively shift to the lower 2θ by varying ψ from 10° to 50° (Fig. 4i, j), indicating the increase in the crystal plane distance d_(012)_ and the presence of tensile stress in the film. In contrast, the SnO_2_/BNH_6_/PVK film exhibits negligible shifts across different angles, suggesting that the d_(012)_ remains nearly constant at different depths of the perovskite film, indicating released residual stress. The slopes of the fitted lines by fitting 2θ as a function of sin^2^ψ and the residual stress are shown in Fig. 4k, l. The SnO_2_/BNH_6_/PVK film exhibited smaller negative slope (− 0.009) and residual stress (4.27 MPa) compared with the negative slope (− 0.092) and residual stress (43.65 MPa) for the SnO_2_/PVK film, suggesting BNH_6_ buried modification can significantly release residual tensile stress in perovskite film, which is favorable for the efficiency and stability of PSCs [53].

**Fig. 5:**
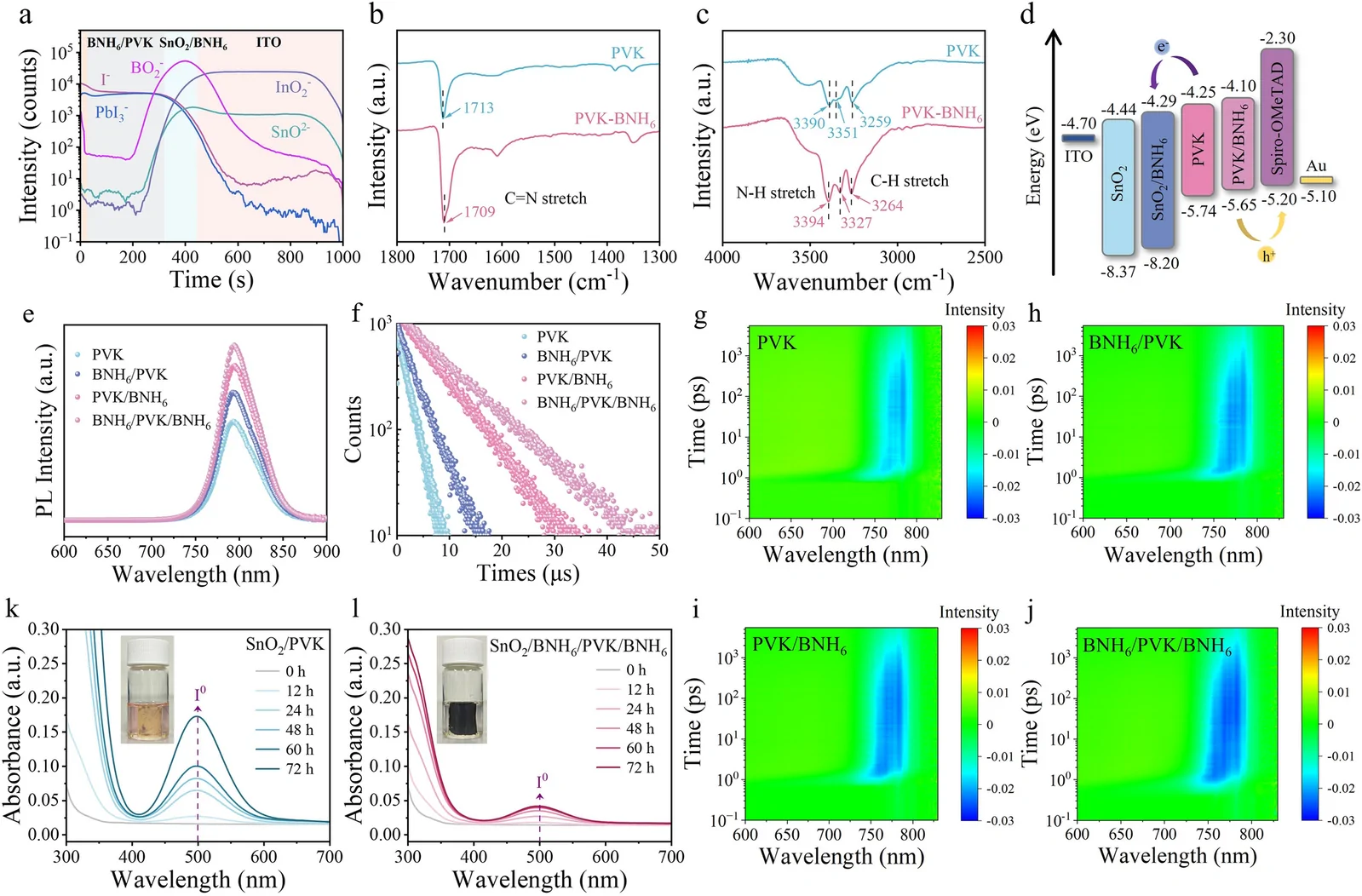
Characteristics of perovskite films. **a** ToF–SIMS depth profiles of I^−^, PbI_3_^−^, InO_2_^−^, SnO^2−^ and BO_2_^−^ for ITO/SnO_2_/BNH_6_/PVK/BNH_6_. **b, c** FTIR spectra of the PVK and PVK-BNH_6_ films. **d** The schematic diagram of the energy level arrangement of the components with and without BNH_6_. **e** PL and **f** TRPL spectra of the PVK, BNH_6_/PVK, PVK/BNH_6_ and BNH_6_/PVK/BNH_6_ films. Characteristic pseudo-color TAS plots of the **g** PVK, **h** BNH_6_/PVK,** i** PVK/BNH_6_ and **j** BNH_6_/PVK/BNH_6_ films. UV–vis spectra of the toluene immersed with the **k** SnO_2_/PVK and **l** SnO_2_/BNH_6_/PVK/BNH_6_ films under light and heat exposure at different times. Inset photographs are the samples after light and heat aging for 72 h

Time-of-flight secondary-ion mass spectrometry (ToF–SIMS) reveals that the emergence of BO_2_^−^ proves BNH_6_ had been successfully introduced into the buried and the upper interfaces of perovskite film (Fig. 5a). FTIR was then applied to study the interactions between BNH_6_ and perovskite. As shown in Fig. 5b, c, the C=N stretching peak shifts from 1713 to 1709 cm^−1^, the N–H stretching peak shifts from 3390 to 3394 cm^−1^, and the C–H stretching peak shifts from 3351/3259 to 3327/3264 cm^−1^ on adding BNH_6_, suggesting the generation of the hydrogen bonds between BNH_6_ and FAI. The interactions between BNH_6_ and perovskite were further validated by XPS (Fig. S20). It is found that Pb 4*f*, I 3*d,* and N 1*s* from the PVK/BNH_6_ film all exhibit certain red-shift compared to the PVK film, further proving the strong interactions between BNH_6_ and FAI/PbI_2_. UPS and LEIPS were employed to illustrate the energy band alignment of perovskite with BNH_6_ modification. Figure S21 exhibits that BNH_6_ treatment decreases the *E*_cutoff_ from 17.00 to 16.92 eV, resulting in the work function of the perovskite film shifts from 4.22 to 4.30 eV. Meanwhile, the valence band (*E*_V_) calculated from UPS shifts from − 5.74 to − 5.65 eV, and the *E*_C_ measured from LEIPS shifts from − 4.25 to − 4.10 eV (Fig. S22), demonstrating better energy alignment between perovskite layer and hole transport layer for extracting holes and blocking electrons (Fig. 5d). UV–vis absorption spectra show that the perovskite films incorporating BNH_6_ exhibit stronger absorption compared with the SnO_2_/PVK film, with SnO_2_/BNH_6_/PVK/BNH_6_ film exhibiting the strongest absorption, which can be attributed to the enhanced film quality after introducing BNH_6_. In addition, the bandgap is determined to be 1.53 eV, which has negligible influence with BNH_6_ modification (Fig. S23). Moreover, Urbach energies (*E*_u_) were also compared from UV–vis absorption spectra (Figs. S24 and S25). The lowest *E*_u_ of SnO_2_/BNH_6_/PVK/BNH_6_ film represents the highest structural quality during the crystal formation of perovskites [54], which is attributed to the multifunctional role of the BNH_6_ molecule in passivating various defects.

Then we recorded photoluminescence (PL), time-resolved photoluminescence (TRPL) and PL mapping to track the film quality and charge recombination dynamics. In order to eliminate the decrease in PL intensity and lifetime caused by SnO_2_ as a transport layer [55, 56], we have prepared the PVK, BNH_6_/PVK, PVK/BNH_6_, BNH_6_/PVK/BNH_6_ films to explore the role of BNH_6_ at the buried and upper interfaces of perovskite. Compared with the PVK film, the BNH_6_/PVK and PVK/BNH_6_ films exhibit enhanced intensity of PL and PL mapping (Figs. 5e and S26), as well as the lifetime (Fig. 5f and Table S3), indicating the suppressed nonradiative recombination with BNH_6_ passivation. Meanwhile, the BNH_6_/PVK/BNH_6_ film modified with dual interfaces exhibits the highest intensity of PL and PL mapping, with the lifetime up to 9.1 µs. These results suggest that the BNH_6_ all-in-one modification strategy can suppress the defect-induced nonradiative recombination to improve the quality of perovskite. Subsequently, femtosecond transient absorption spectroscopy (fs-TAS) was applied to determine the charge transfer behaviors of the four perovskite films mentioned above. The characteristic pseudo-color TAS plots (Fig. 5g–j) show that the BNH_6_/PVK/BNH_6_ film exhibits the least faded ground state bleaching (GSB) signal, as well as the strongest GSB peak intensity among the four samples (Fig. S27), which represent the longest carrier lifetime. This result agrees with the lifetimes gained from the GSB decay kinetic of fs-TAS at 783 nm (Fig. S28) [57–60]. The longer carrier lifetime after BNH_6_ all-in-one modificated implies the trap state elimination and suppressed nonradiative recombination, which is beneficial for the performance of device.

We further investigated the stability of perovskite films. After immersing the perovskite films into toluene solution while simultaneous exposing them to light exposure and applying 60 °C thermal treatment for 72 h, the control film shows noticeable yellowing and significant degradation. The related toluene solution turns pink as well as a strong I^0^ characteristic absorption peak appears at ≈ 500 nm in the UV–vis spectrum (Fig. 5k, l). By comparison, the SnO_2_/BNH_6_/PVK/BNH_6_ film exhibits no significant color change, and the I^0^ characteristic absorption peak is much weaker compared to the SnO_2_/PVK film, indicating that BHN_6_ significantly suppressed the oxidation of I^−^ to I^0^ and the formation of I^0^ and V_I_ defects. In addition, XRD results (Fig. S29) show that after accelerated aging, the I^0^ characteristic peak intensity of the control film is much higher than that of the target film, further proving that the improved stability of perovskite films with BNH_6_ all-in-one modification, as well as BNH_6_ can effectively prevent the oxidation of I^−^ to I^0^ and reduce the formation of related defects.

### Device Performance and Characterization

**Fig. 6:**
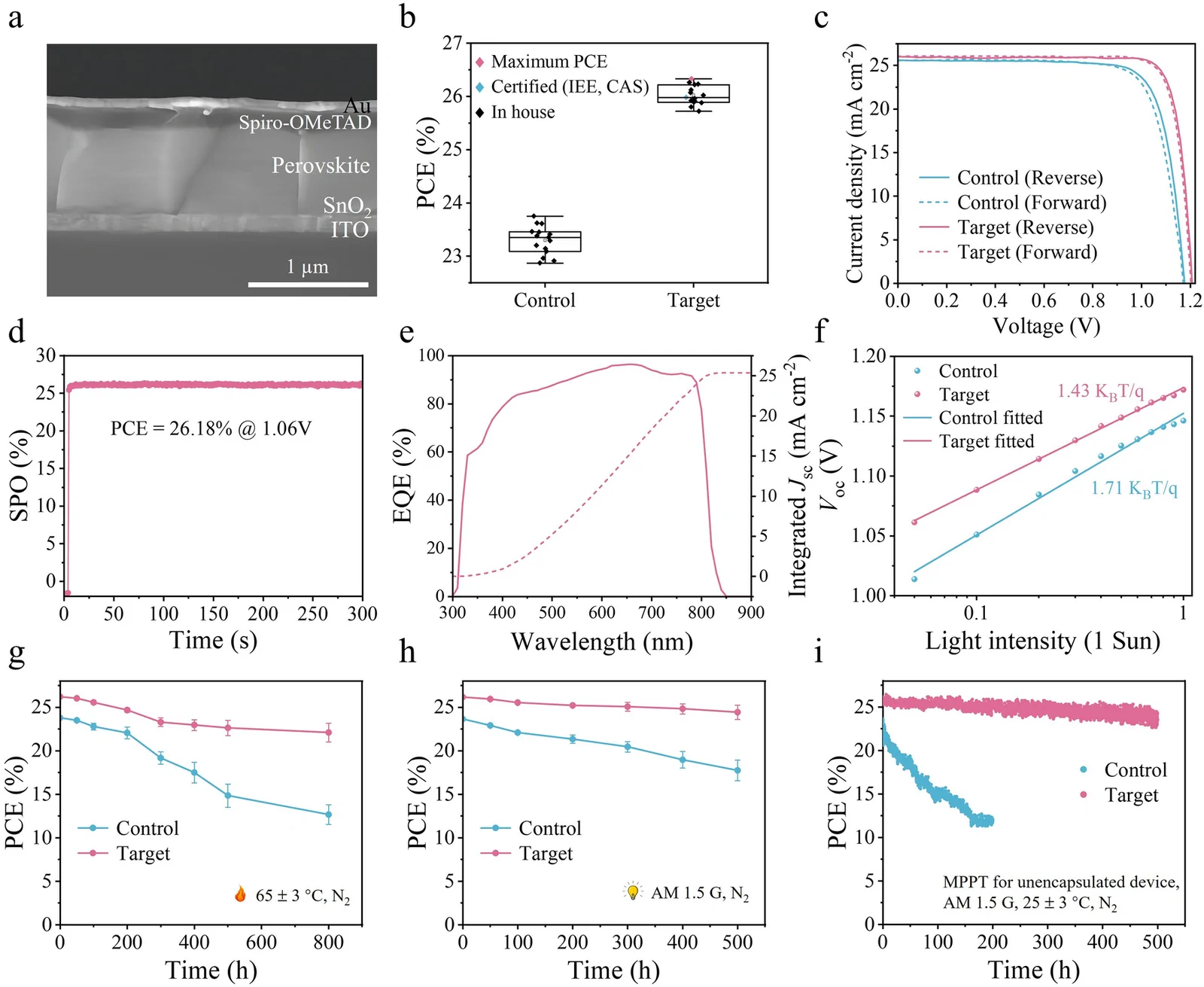
Performance and stability of the devices. **a** Cross-sectional SEM image of the device structure. **b** Efficiency statistics of both solar cells including the certified value obtained at the Institute of Electrical Engineering Chinese Academy of Sciences (IEE, CAS) and lab measured efficiencies. **c** J−V curves of the control and target devices. **d** SPO curve of the target device. **e** EQE curve and integrated current density of the target device. **f** Dependence of *V*_oc_ on light intensity of the control and target devices. Long-term stability of the unencapsulated control and target devices stored **g** in glove box with N_2_ atmosphere under 65 ± 3 °C heat, **h** in glove box with N_2_ atmosphere under AM 1.5 G illumination. **i** MPPT values recorded for the control and target devices with unencapsulation under AM 1.5 G illumination at 25 ± 3 °C with N_2_ atmosphere

To evaluate the impact of BNH_6_ all-in-one modification strategy on photovoltaic performance, we fabricated a typical *n-i-p* PSC with the architecture of glass/ITO/SnO_2_/Cs_0.05_FA_0.95_PbI_3_/Spiro-OMeTAD/Au (Fig. 6a). We recorded the *J* − *V* characteristics under simulated 1 sun irradiation with an intensity of 100 mW cm^−2^ (AM 1.5 spectrum). In order to regulate the concentrations of BNH_6_ at the buried and upper interfaces of perovskite film separately, we made devices under each condition for comparison, as shown in Fig. S30 and Table S4. After optimization, the best concentrations of BNH_6_ are both 1 mg mL^−1^ at the buried and upper interfaces of perovskite film. Thus, 15 PSCs were prepared under control and target (BNH_6_ all-in-one modification) conditions to study the statistical distribution of current density (*J*_sc_), *V*_oc_, FF and PCE (Figs. 6b and S31). By contrast, the average PCE of target is higher than that of control. In addition, the champion PCE of the target PSCs significantly increases from 23.62% to 26.43% (the pink dot in Fig. 6b), with the *V*_oc_ of 1.206 V, *J*_sc_ of 26.05 mA cm^−2^, and FF of 84.12%, showing a negligible hysteresis (Fig. 6c and Table S5). The significant improvement in PCE can be attributed to the reduction of I^0^ related defects and the passivation of the buried and upper interfaces of perovskite film. Some devices were sent to an independent photovoltaic testing laboratory (Institute of Electrical Engineering, Chinese Academy of Sciences) for certification, with a certified PCE of 25.98% (the blue dot in Fig. 6b, certificate attached in Fig. S32). Additionally, the device exhibits a high stabilized power output (SPO) of 26.18% (biased at 1.06 V), as shown in Fig. 6d. Meanwhile, Fig. 6e shows the external quantum efficiency (EQE) spectrum, with the integrated *J*_sc_ of 25.36 mA cm^−2^ for the target device, which is closed to the *J*_sc_ determined from the *J* − *V* curves. The analysis of EQE spectrum shows that the bandgap of perovskite is about 1.53 eV (Fig. S33), which is consistent with the bandgap measured from UV–vis absorption spectrum (Fig. S23).

We further utilized space charge-limited current (SCLC) to measure the trap density of the perovskite films (Fig. S34 and Table S6). In electron-only devices (ITO/SnO_2_/perovskite/PCBM/Ag), the trap density drops from 2.79 × 10^15^ cm^−3^ for the SnO_2_/PVK device to 1.59 × 10^15^ cm^−3^ for the SnO_2_/BNH_6_/PVK device. In hole-only devices (ITO/PEDOT:PSS/perovskite/Spiro-OMeTAD/Au, the trap densities are reduced from 1.77 × 10^15^ cm^−3^ for the PVK/Spiro device to 1.29 × 10^15^ cm^−3^ for the PVK/BNH_6_/Spiro device. These results confirm that BNH_6_ all-in-one modification effectively reduces the trap density in perovskites, which is crucial for minimizing nonradiative recombination and improving device performance. Electrical impedance spectroscopy (EIS) results (Fig. S35 and Table S7) further demonstrate that the target device shows larger recombination resistance (R_rec_), suggesting the charge recombination was greatly suppressed with BNH_6_ all-in-one modification. This is further corroborated by transient photovoltage (TPV) measurement (Fig. S36), where the lifetime of the target device increases to 69.57 µs compared to 57.72 µs for the control device, which reflects the efficiency of BNH_6_ treatment. Capacitance–voltage (*C* − *V*) measurement (Fig. S37) shows that the built-in electric field (*V*_bi_) increases from 1.01 to 1.09 V with BNH_6_ modification. The enhanced *V*_bi_ promotes better charge separation and carrier transport, contributing to the improved *V*_oc_ and FF in PSCs. Besides, the *V*_oc_ variation of PSCs under different light intensities shows that the slope reduced from 1.71 $k_{B} T/q$ for the control device to 1.43 $k_{B} T/q$ for the target device (Fig. 6f). The lower slope value of the target device indicates a decrease in trap-assisted recombination in PSCs, further supporting the effectiveness of BNH_6_ in enhancing charge transport and device performance.

The unencapsulated devices under thermal conditions and illumination were evaluated according to the Organic Photovoltaic Stability (ISOS) protocols [61]. Devices aged under thermal stress (65 ± 3 °C, N_2_ atmosphere) were investigated to monitor the heat stability (ISOS-T-1, Fig. 6g). After continuous heating for around 800 h, the target devices still maintain 84% of the initial PCE, which is remarkably higher than that of the control devices (53%). Furthermore, the light stability was investigated under continuous white LED illumination (100 mV cm^−2^) in N_2_ atmosphere for 500 h (ISOS-L-1, Fig. 6h). The target devices preserve 93% of the initial PCE, which is higher than the 75% for the control devices. Meanwhile, the heat and light stability were further researched by XRD to track the degradation of the control and target films (Fig. S38). The control film exhibits distinct degradation after 500 h, as evidenced by the increased intensity of PbI_2_ peak and the appearance of δ-FAPbI_3_ peak, whereas the target film shows lower intensity of PbI_2_ peak and no obvious undesired peaks. In addition, we have evaluated the stability of the unencapsulated devices under ambient air conditions (50–60% RH, 25 °C) (ISOS-D-1, Fig. S39). After 500 h, the control device retained only 85% of the initial PCE, whereas the target device maintained 96% of the initial PCE. These results further demonstrate the effectiveness of the all-in-one modification strategy under more practical conditions.

The operational stability of PSCs was also examined by maximum power point tracking (MPPT) under AM 1.5 G illumination at 25 ± 3 °C with N_2_ atmosphere (ISOS-L-1, Fig. 6i). After continuous monitoring for 500 h, the target device displays outstanding stability, maintaining 90% of the initial PCE, while the control device only maintains 51% of the initial PCE after 200 h. It suggests that the defects caused by ion migration and organic component volatilization in the target device have been significantly suppressed. To further validate the stability of BNH_6_, we conducted chemical stability tests on both BNH_6_ powders and SnO_2_/BNH_6_ films under accelerated aging conditions (AM 1.5 G illumination at 25 ± 3 °C with N_2_ atmosphere, 7 days), as shown in Figs. S40 and S41. The results demonstrate excellent chemical stability, with no significant changes observed in the characteristic signals after aging. These findings confirm that the incorporation of BNH_6_ does not induce chemical or electrical degradation. Moreover, as shown in Fig. S42, the contact angle for the control film is 53.8°, which increases to 75.9° for the target film, implying the improved hydrophobicity of perovskite film. These results strongly indicate that the PSCs exhibit improved device performance via BNH_6_ all-in-one modification.

## Conclusion

In summary, an all-in-one modification strategy was proposed by introducing the multifunctional complex BNH_6_ to simultaneously address critical challenges at both the buried and upper interfaces of perovskite film. This approach enables BNH_6_ to uniquely realize dual-interfacial defect passivation and iodide oxidation suppression by interacting with SnO_2_ through hydrolysis, coordinating with Pb^2+^ and inhibiting the oxidation of I^−^. These synergistic features enhance perovskite film quality, lead to better energy level alignment, reduce defects, and extend carrier lifetime. As a result, the BNH_6_ all-in-one modification strategy significantly improves the PCE to 26.43%. Furthermore, the BNH_6_-modified devices exhibit enhanced operational stability, preserving 90% of the initial PCE after continuous tracking for 500 h, a bright contrast to 51% of the control device for 200 h. Thus, the all-in-one strategy streamlines device fabrication by eliminating the need for separate interface treatments, offering a scalable and practical pathway toward high-performance perovskite photovoltaics for practical applications.

## Supplementary Information

Below is the link to the electronic supplementary material.

Supplementary file1 (DOCX 13164 KB)
