# Supplementary material for: Ammonia Borane All-In-One Modification Strategy Enables High-Performance Perovskite Solar Cells
Source: Nanomicro Lett. 2026 Jan 2;18:93. doi: 10.1007/s40820-025-01951-6 (PMC12757506; doi:10.1007/s40820-025-01951-6)
Supplement: Supplementary file 1 — Supplementary file1 (DOCX 13164 KB) [file 40820_2025_1951_MOESM1_ESM.docx]

Supporting Information for

**Ammonia Borane** **All-In-One Modification Strategy Enables High-Performance Perovskite Solar Cells**

Jiaxin Ma^1,2^, Cong Shao^1,2^, Yirong Wang^2,3^, Guosheng Niu^1,2^, Kaiyi Yang^1,2^, Yao Zhao^4^, Fuyi Wang^2,4^, Zongxiu Nie^2,4^, and Jizheng Wang^1,2^*

^1^ Beijing National Laboratory for Molecular Sciences CAS Key Laboratory of Organic Solids, Institute of Chemistry, Chinese Academy of Sciences, Beijing 100190, P. R. China

^2^ School of Chemical Sciences, University of Chinese Academy of Sciences, Beijing 100049, P. R. China

^3^ CAS Key Laboratory of Engineering Plastics, Institute of Chemistry, Chinese Academy of Sciences, Beijing, P. R. China

^4^ Beijing National Laboratory for Molecular Sciences, National Centre for Mass Spectrometry in Beijing, CAS Key Laboratory of Analytical Chemistry for Living Biosystems, Chinese Academy of Sciences, Beijing, P. R. China

* Corresponding author. E-mail: [jizheng@iccas.ac.cn](mailto:jizheng@iccas.ac.cn) (Jizheng Wang)

**Supplementary Figures and Tables**

$${NH}_{3}\cdot{BH}_{3}+2H_{2}O\to{{NH}_{4}}^{+}+{{BO}_{2}}^{-}+3H_{2}$$

**Fig. S1** The hydrolysis chemical equation of ammonium borane.





**Fig. S2** O 1s XPS spectra of the SnO_2_ and SnO_2_/BNH_6_ films after oxygen plasma treatment





**Fig. S3** *J-V* curves of the devices with no treatment or post treatment by oxygen plasma


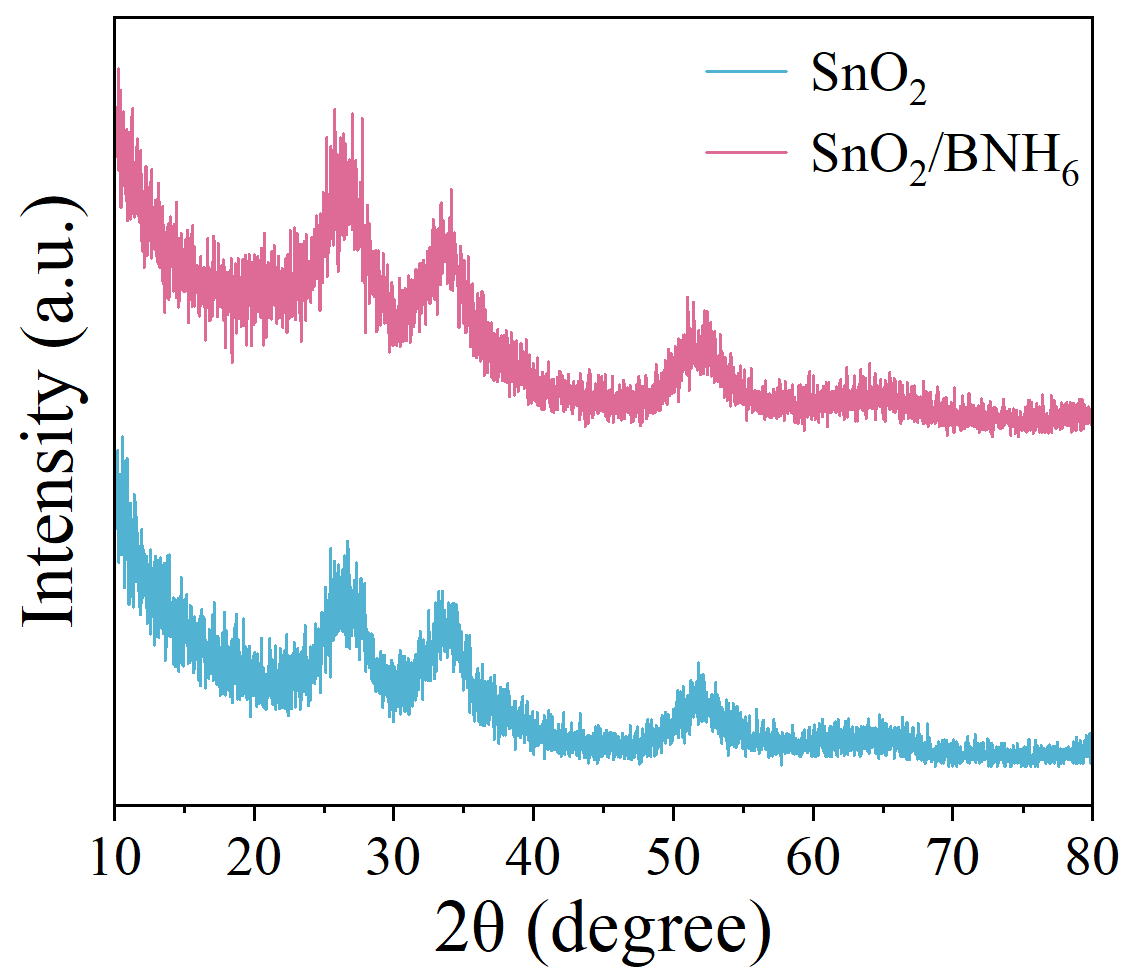


**Fig. S4** XRD spectra of the SnO_2_ and the SnO_2_/BNH_6_ films


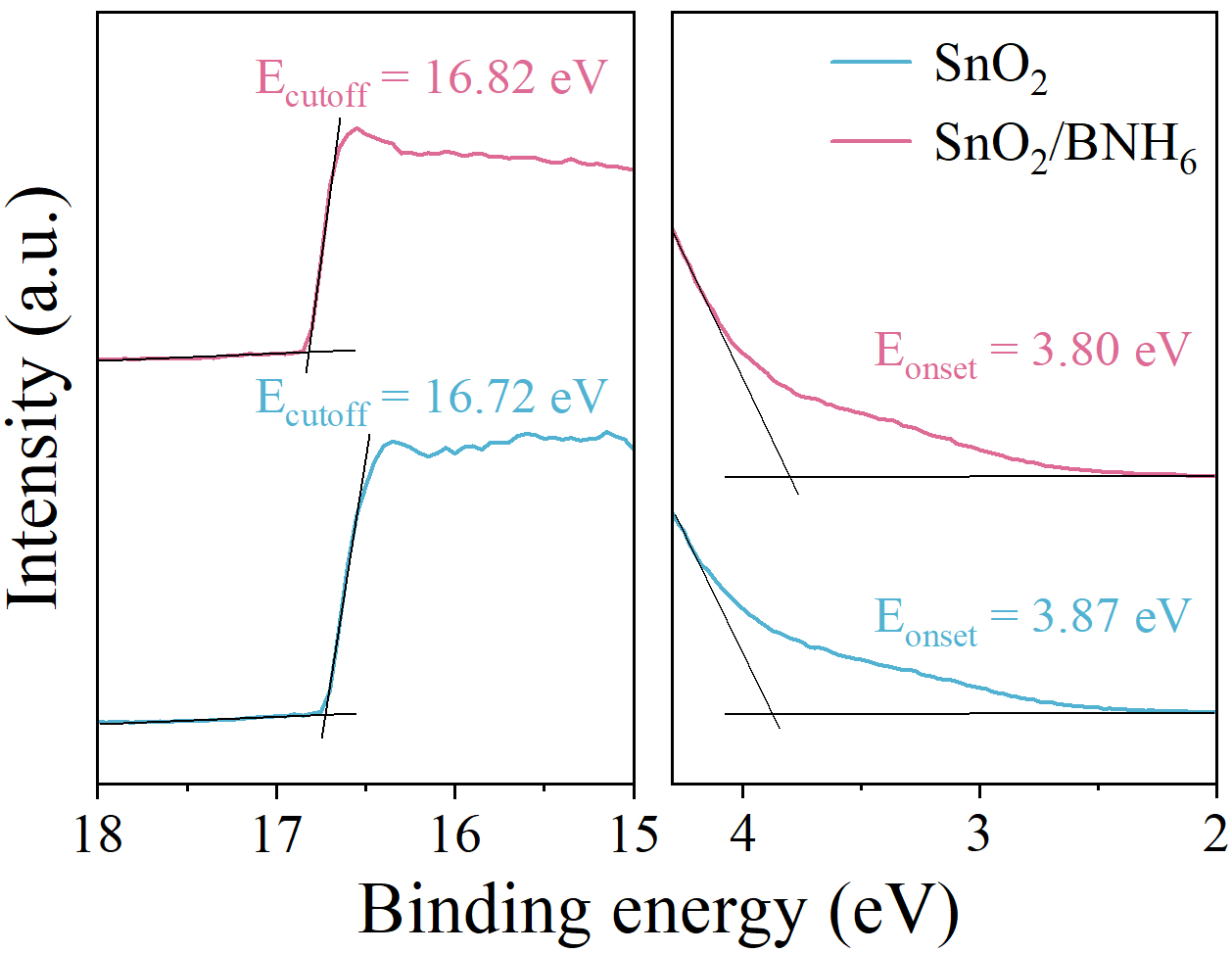


**Fig.** **S5** UPS spectra of the SnO_2_ and SnO_2_/BNH_6_ films


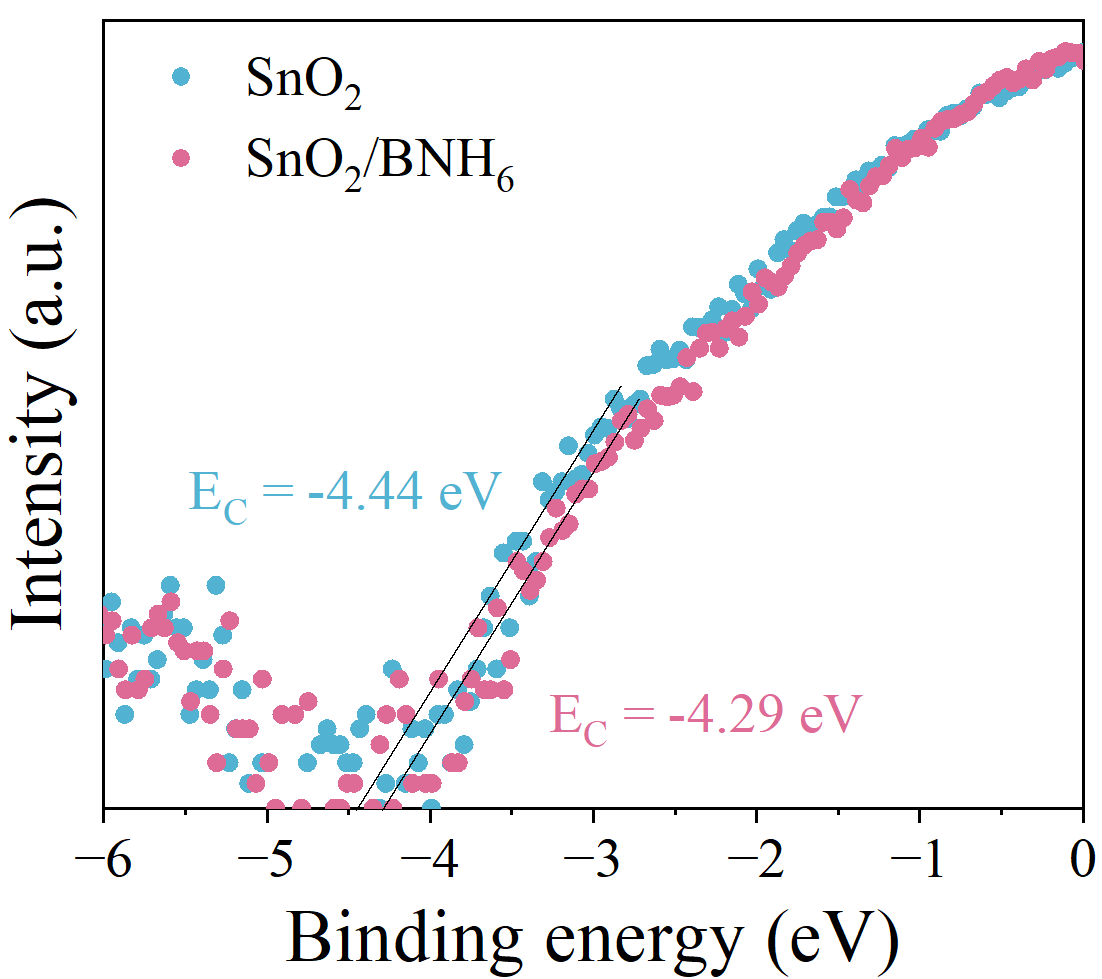


**Fig. S6** LEIPS spectra of the SnO_2_ and SnO_2_/BNH_6_ films


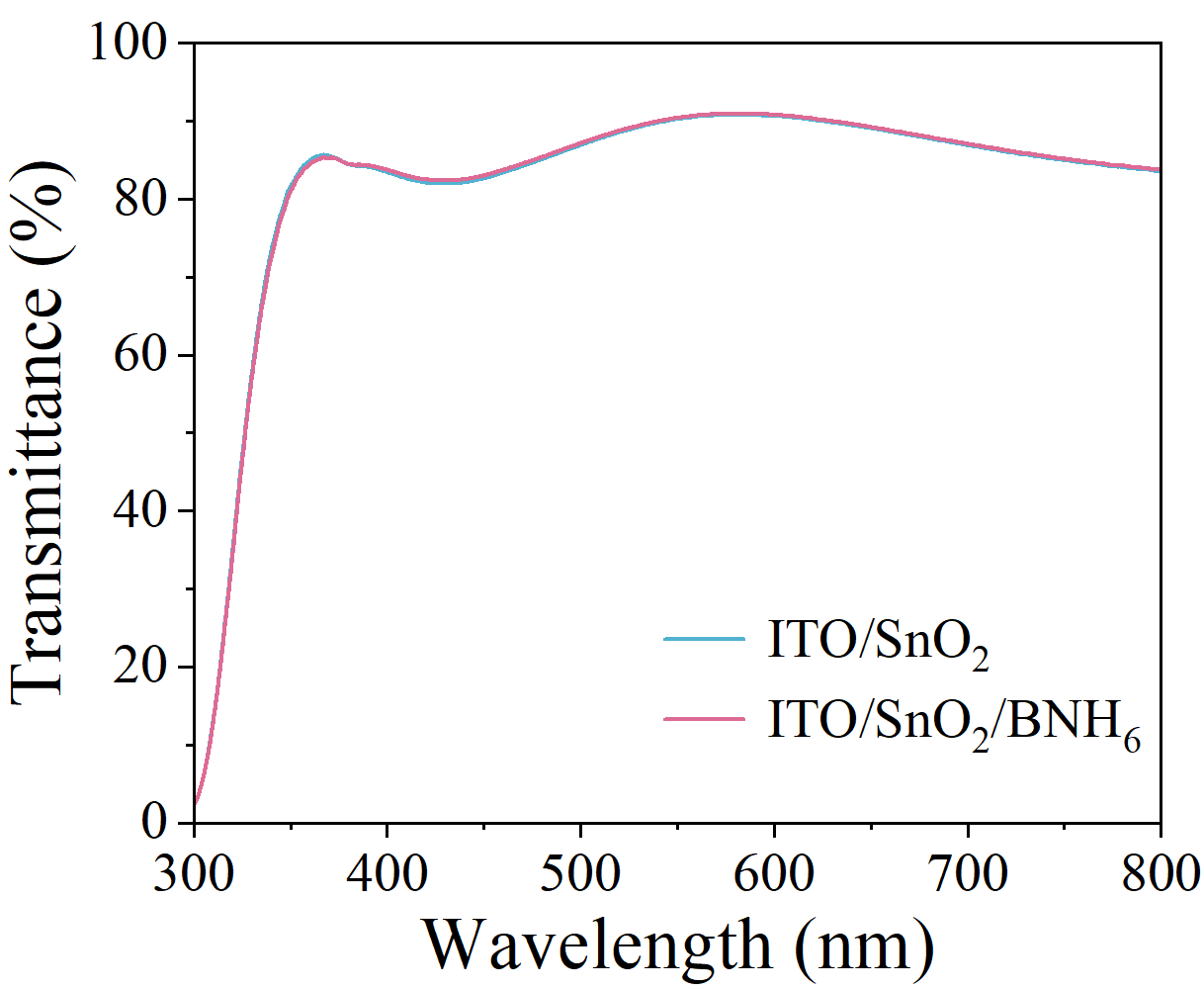


**Fig. S7** The transmittance spectra of the SnO_2_ and SnO_2_/BNH_6_ films


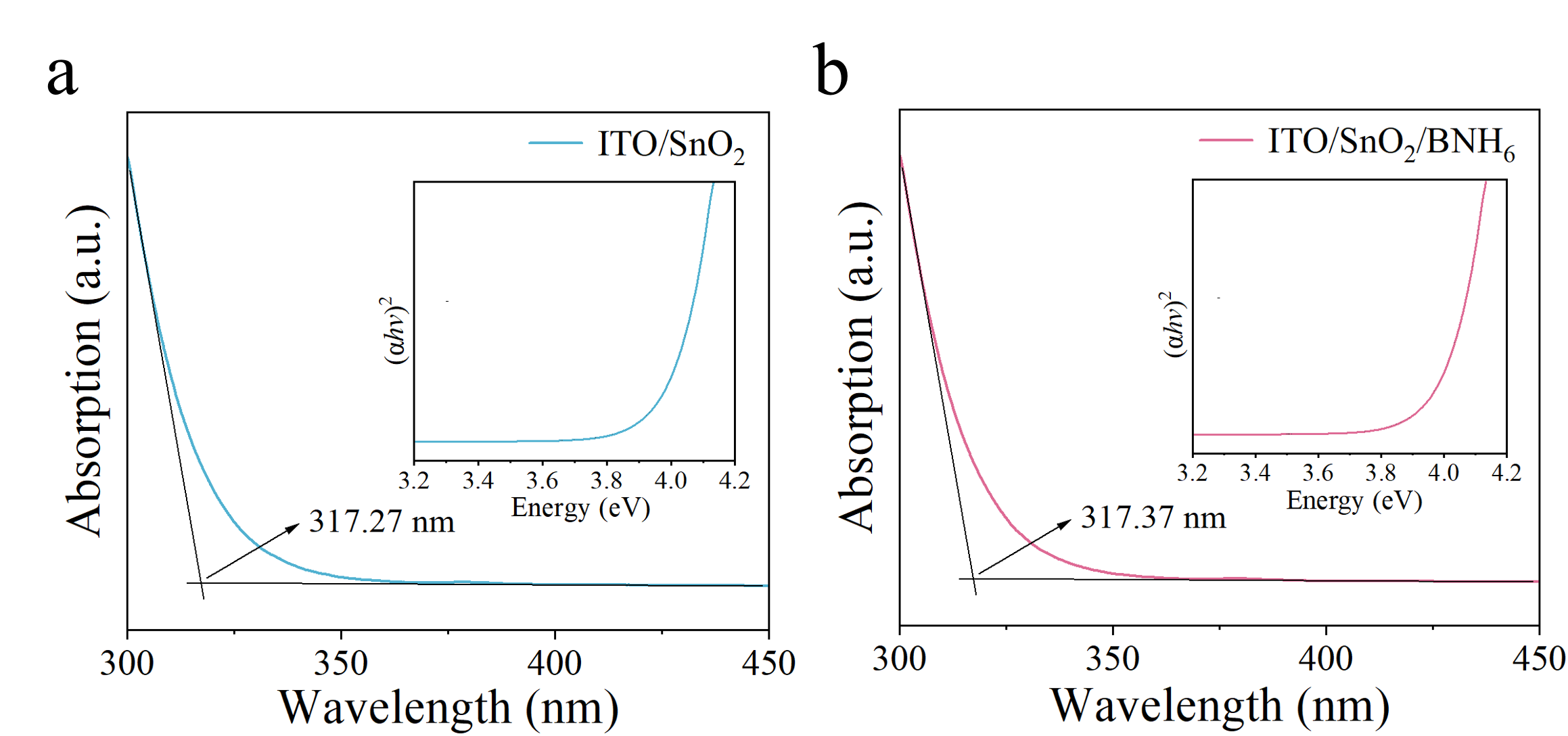


**Fig. S8** UV-vis absorption spectra of the (**a**) SnO_2_ and (**b**) SnO_2_/BNH_6_ films, the insets are Tauc plots of corresponding films


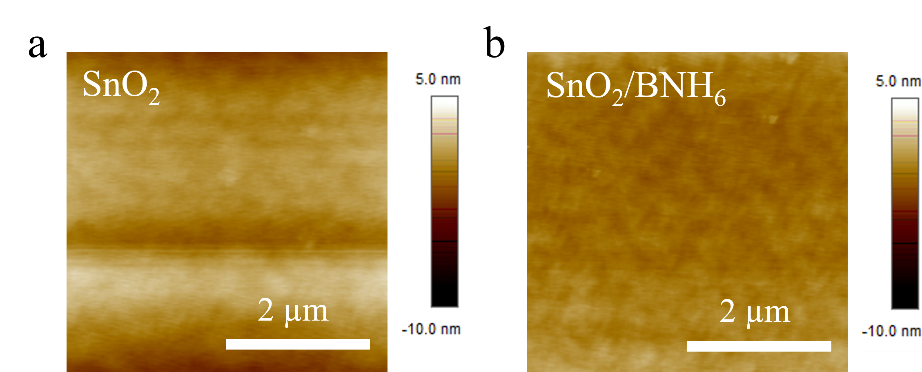


**Fig. S9** AFM images of the (**a**) SnO_2_ film and (**b**) SnO_2_/BNH_6_ film


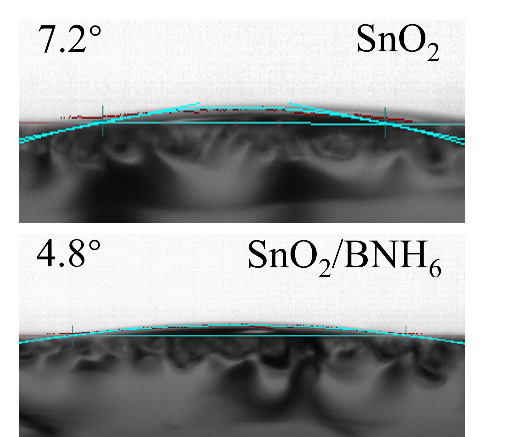


**Fig. S10** Contact angles of perovskite precursor solutions on the SnO_2_ and SnO_2_/BNH_6_ films


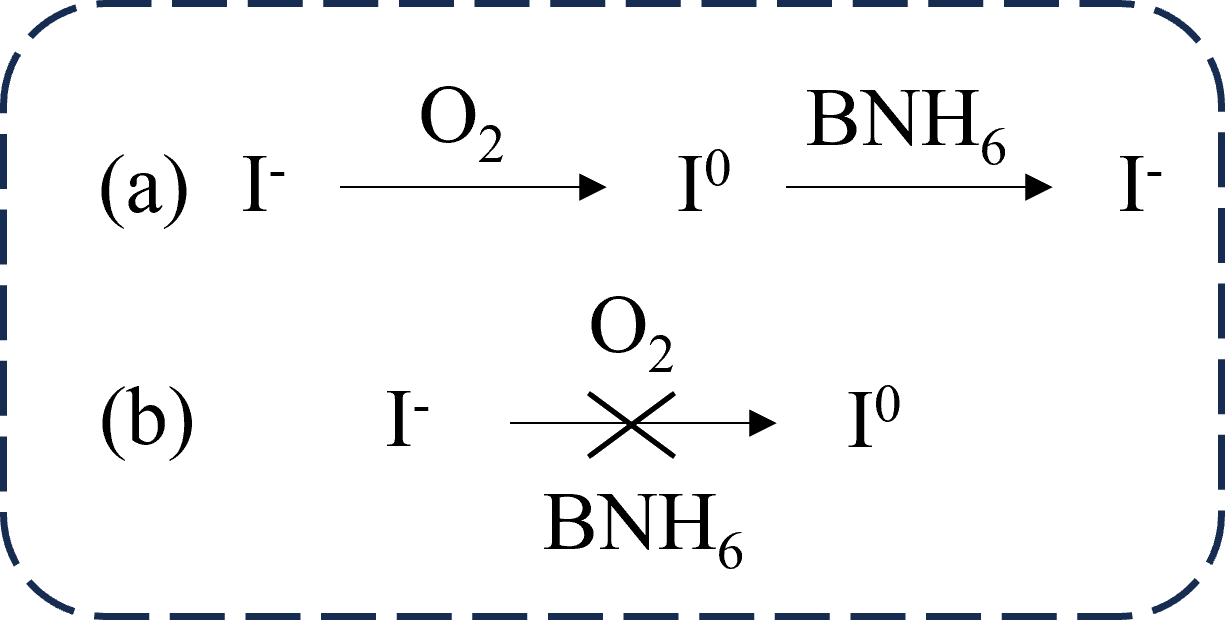


**Fig. S11** Chemical reaction expression of I^0^ and I^-^





**Fig. S12** Ion chromatography of the concentrations of I^-^ at different times with and without BNH_6_


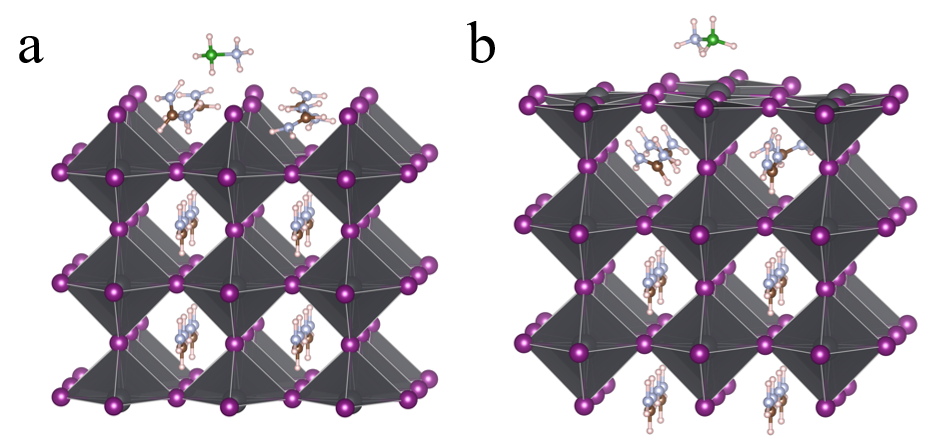


**Fig. S13** Supercell constructed for DFT calculations, in which BNH_6_ molecules are adsorbed on the FAPbI_3_ (001) surface via (**a**) FAI and (**b**) PbI_2_ terminations, respectively


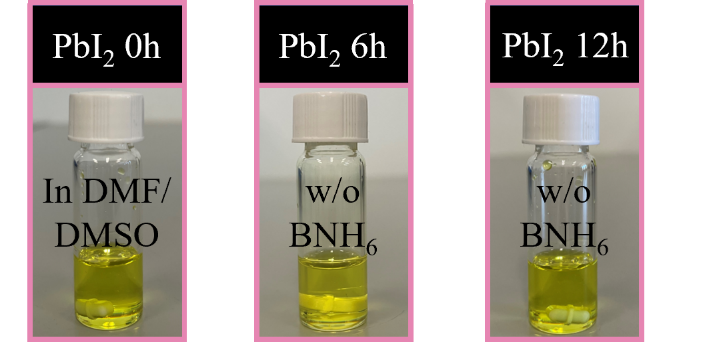


**Fig. S14** The color change process of pristine PbI_2_ solution





**Fig. S15** FTIR spectra of PbI_2_, BNH_6_, and PbI_2_ with BNH_6_ films


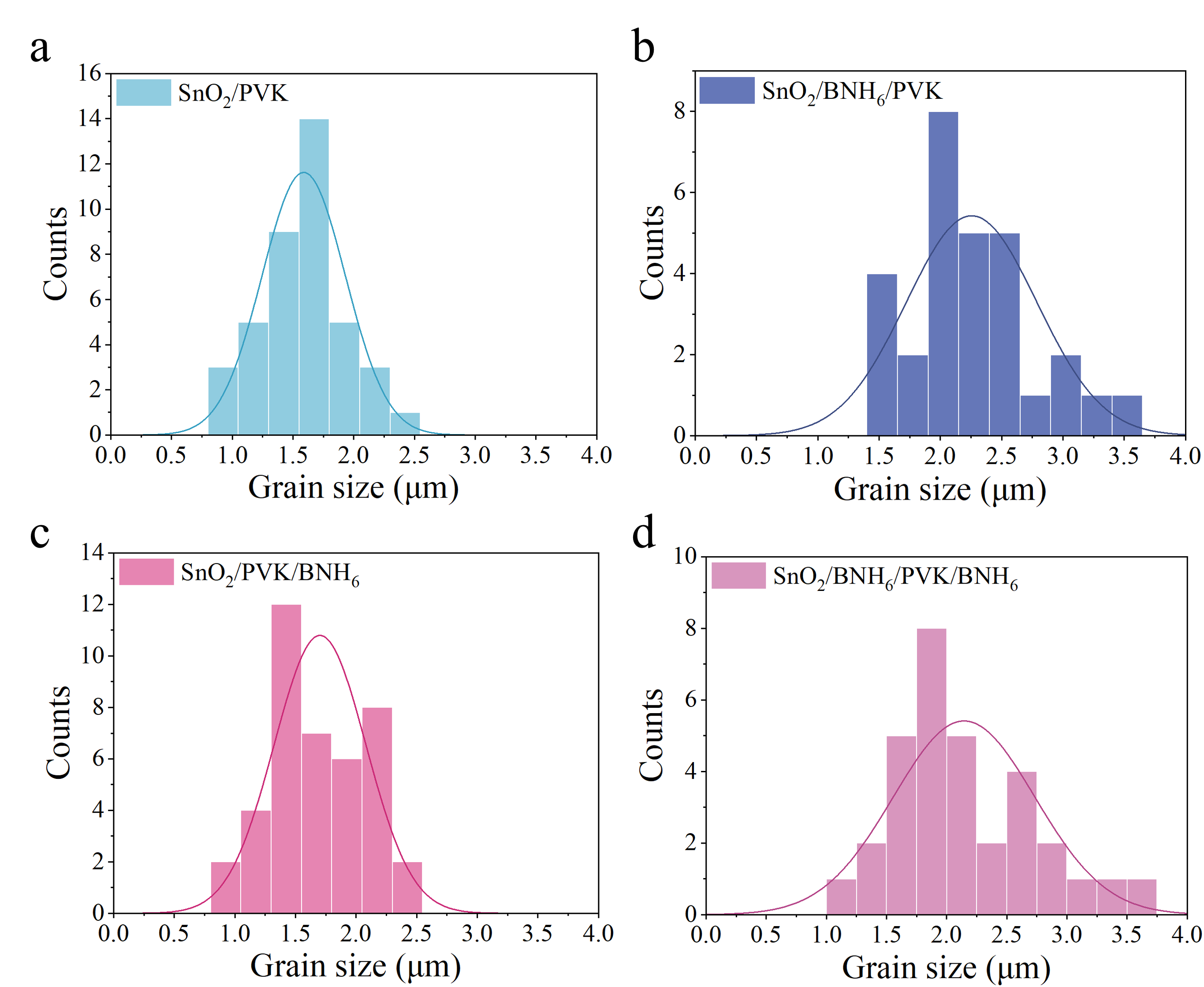


**Fig. S16** Distribution histogram of crystal grain sizes of the perovskite films: (**a**) SnO_2_/PVK, (**b**) SnO_2_/BNH_6_/PVK, (**c**) SnO_2_/PVK/BNH_6_ and (**d**) SnO_2_/BNH_6_/PVK/BNH_6_


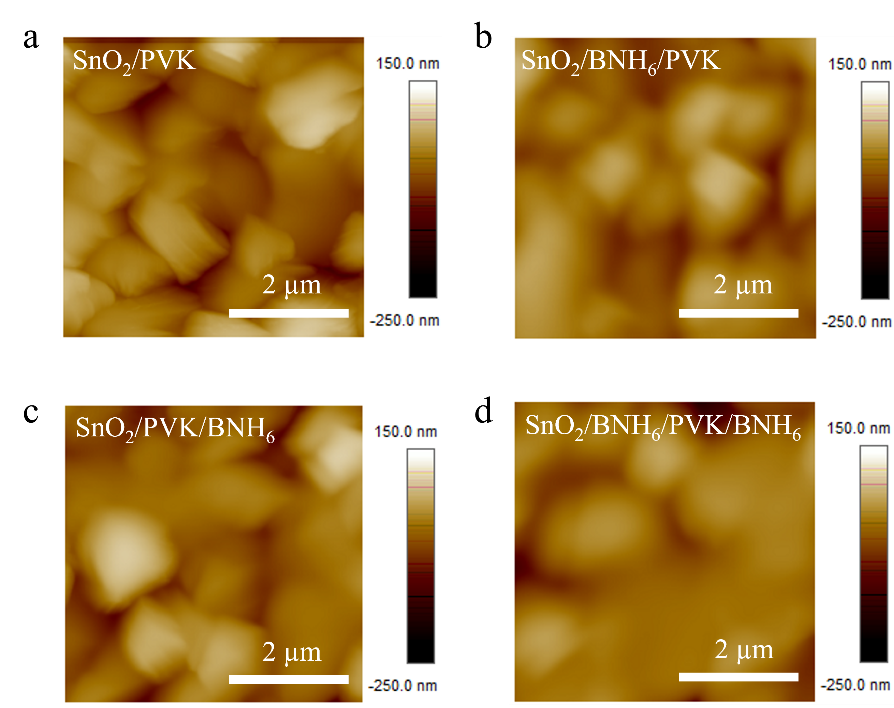


**Fig. S17** AFM surface images of the perovskite films: (**a**) SnO_2_/PVK, (**b**) SnO_2_/BNH_6_/PVK, (**c**) SnO_2_/PVK/BNH_6_, and (**d**) SnO_2_/BNH_6_/PVK/BNH_6_


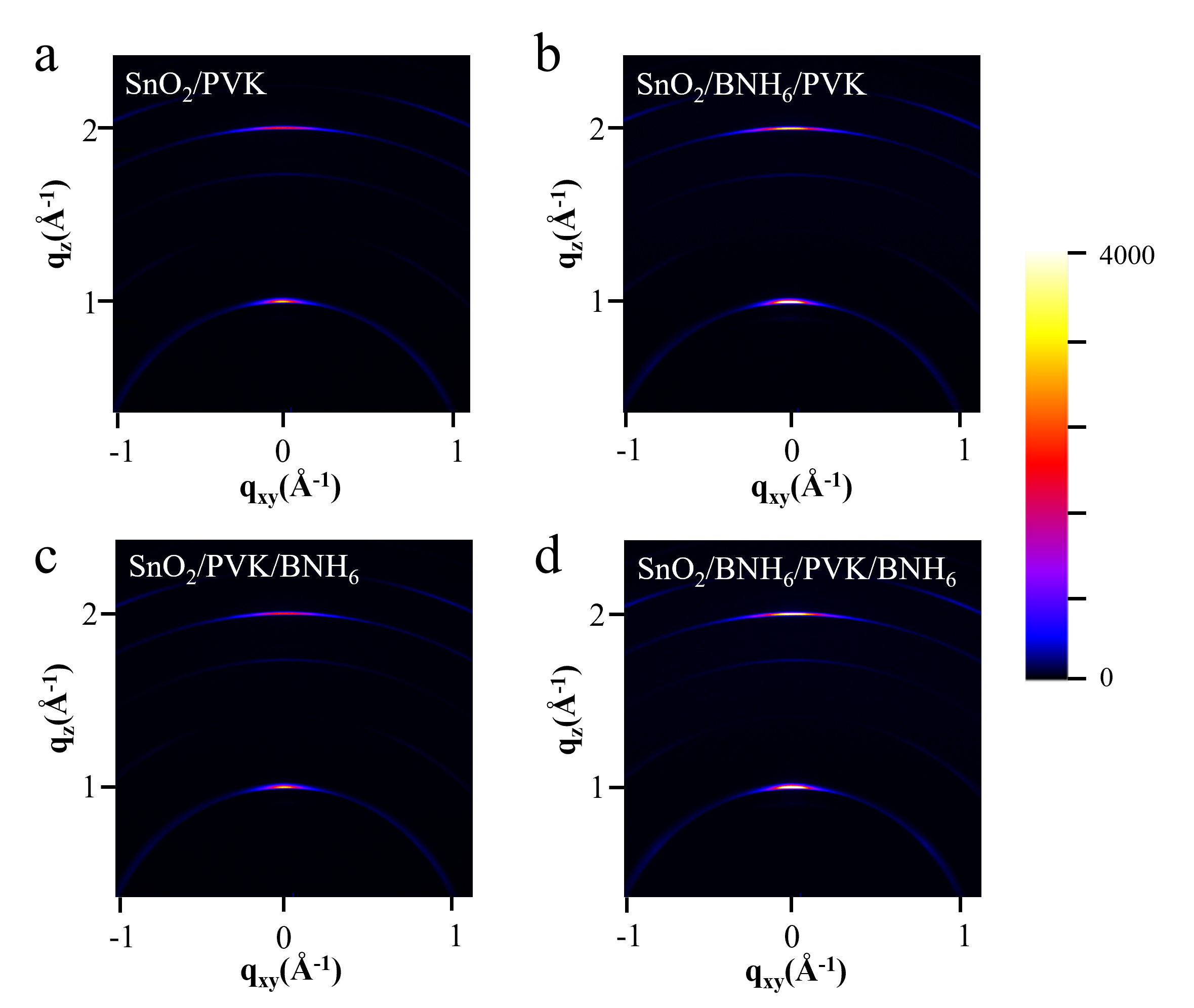


**Fig. S18** 2D-XRD images of the perovskite films: (**a**) SnO_2_/PVK, (**b**) SnO_2_/BNH_6_/PVK, (**c**) SnO_2_/PVK/BNH_6_, and (**d**) SnO_2_/BNH_6_/PVK/BNH_6_


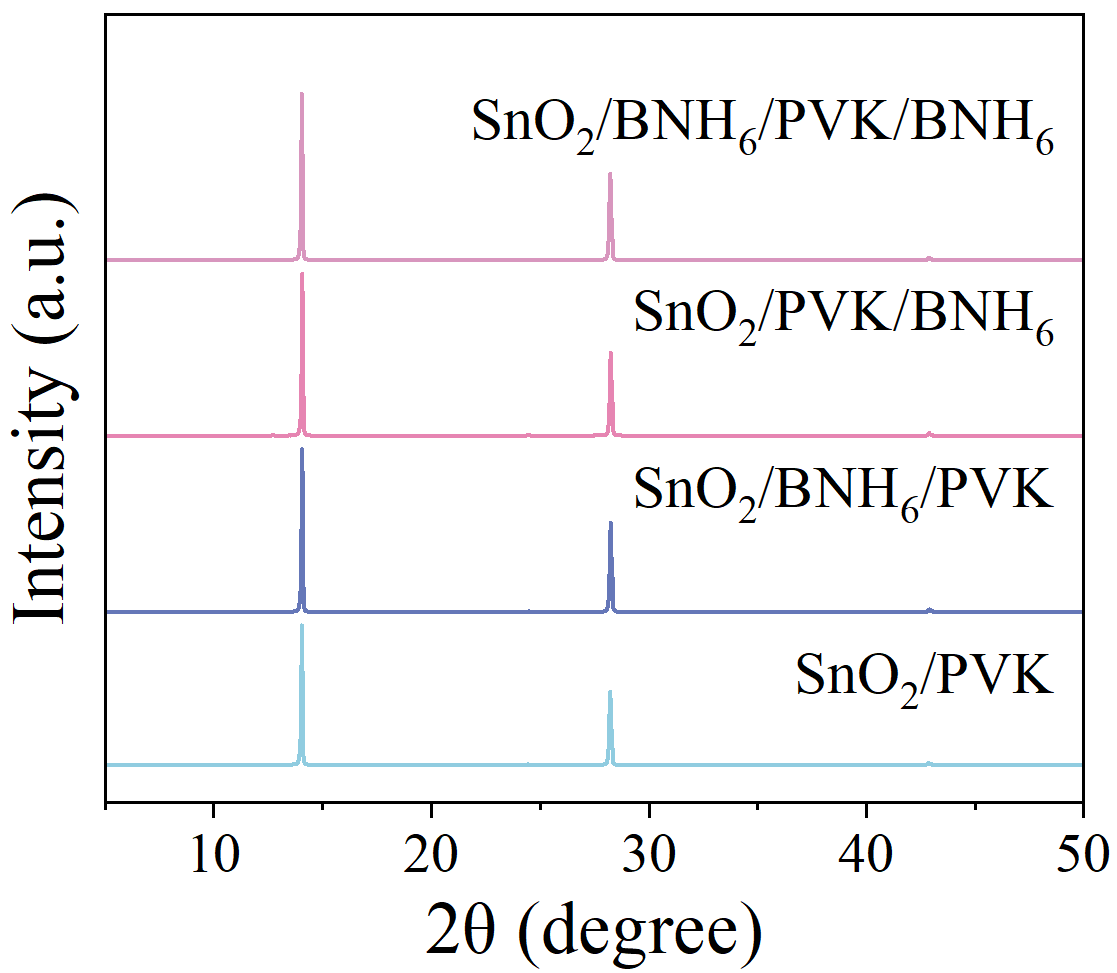


**Fig. S19** XRD images of the SnO_2_/PVK, SnO_2_/BNH_6_/PVK, SnO_2_/PVK/BNH_6_ and SnO_2_/BNH_6_/PVK/BNH_6_ films


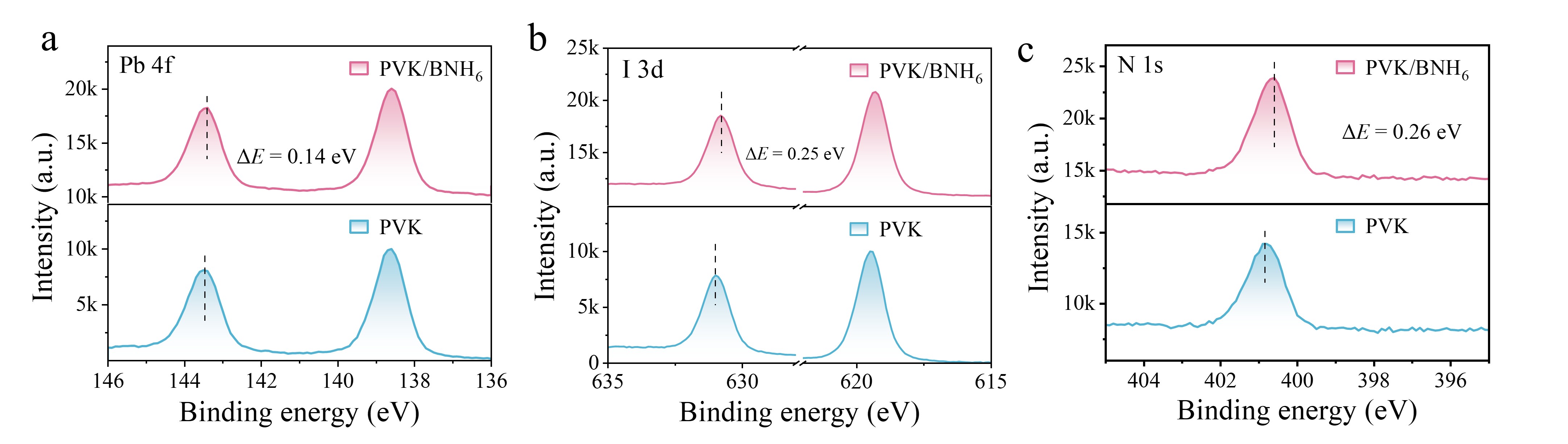


**Fig. S20** XPS spectra of the PVK and PVK/BNH_6_ films. (**a**) Pb 4f, (**b**) I 3d, (**c**) N 1s


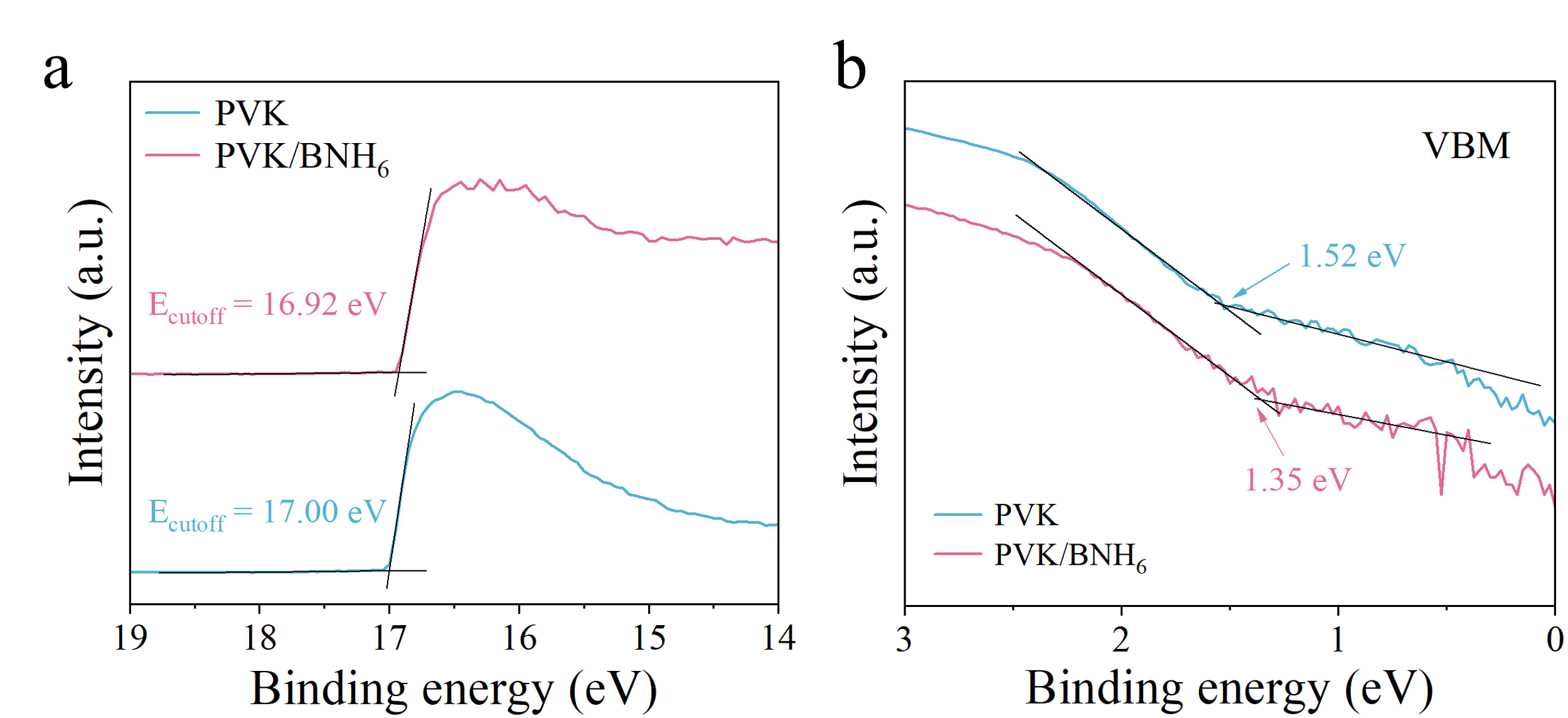


**Fig. S21** UPS spectra of the PVK and PVK/BNH_6_ films


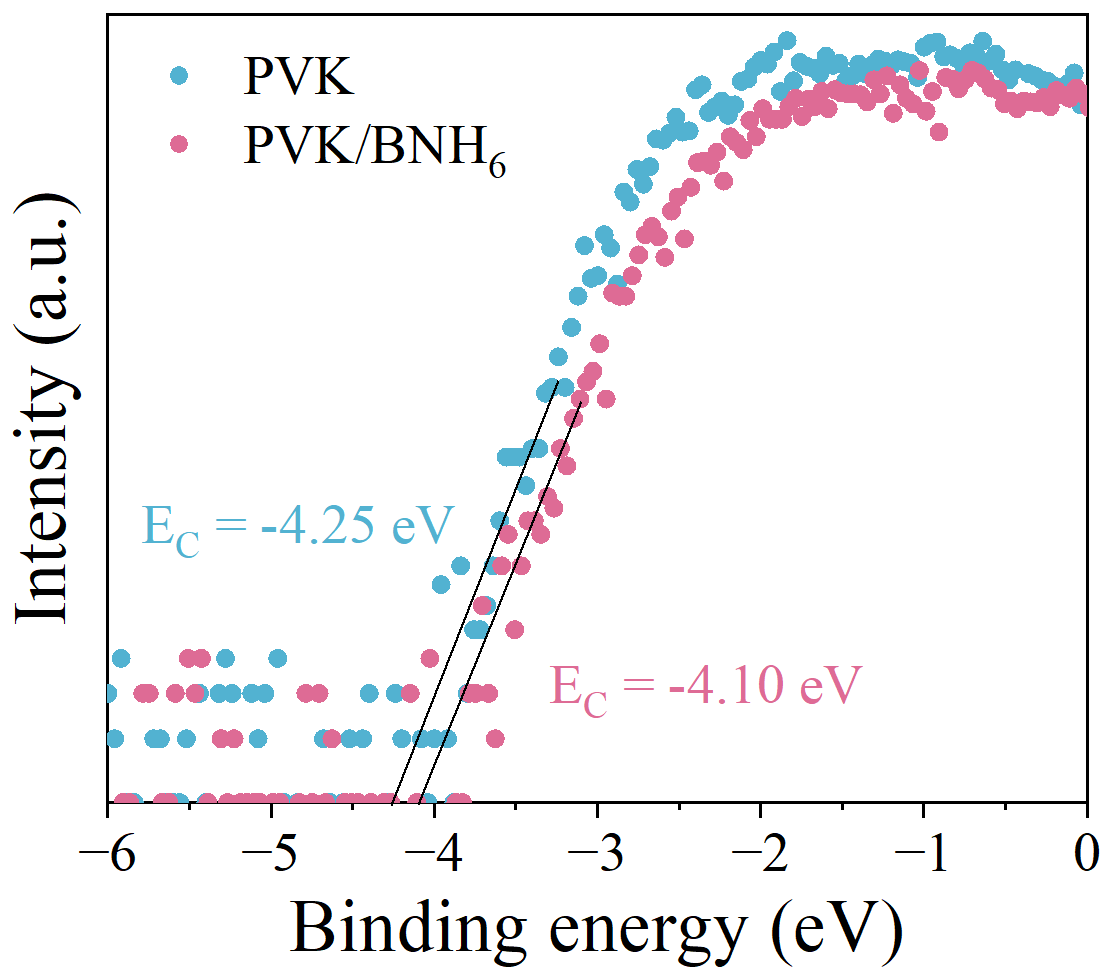


**Fig. S22** LEIPS spectra of the PVK and PVK/BNH_6_ films


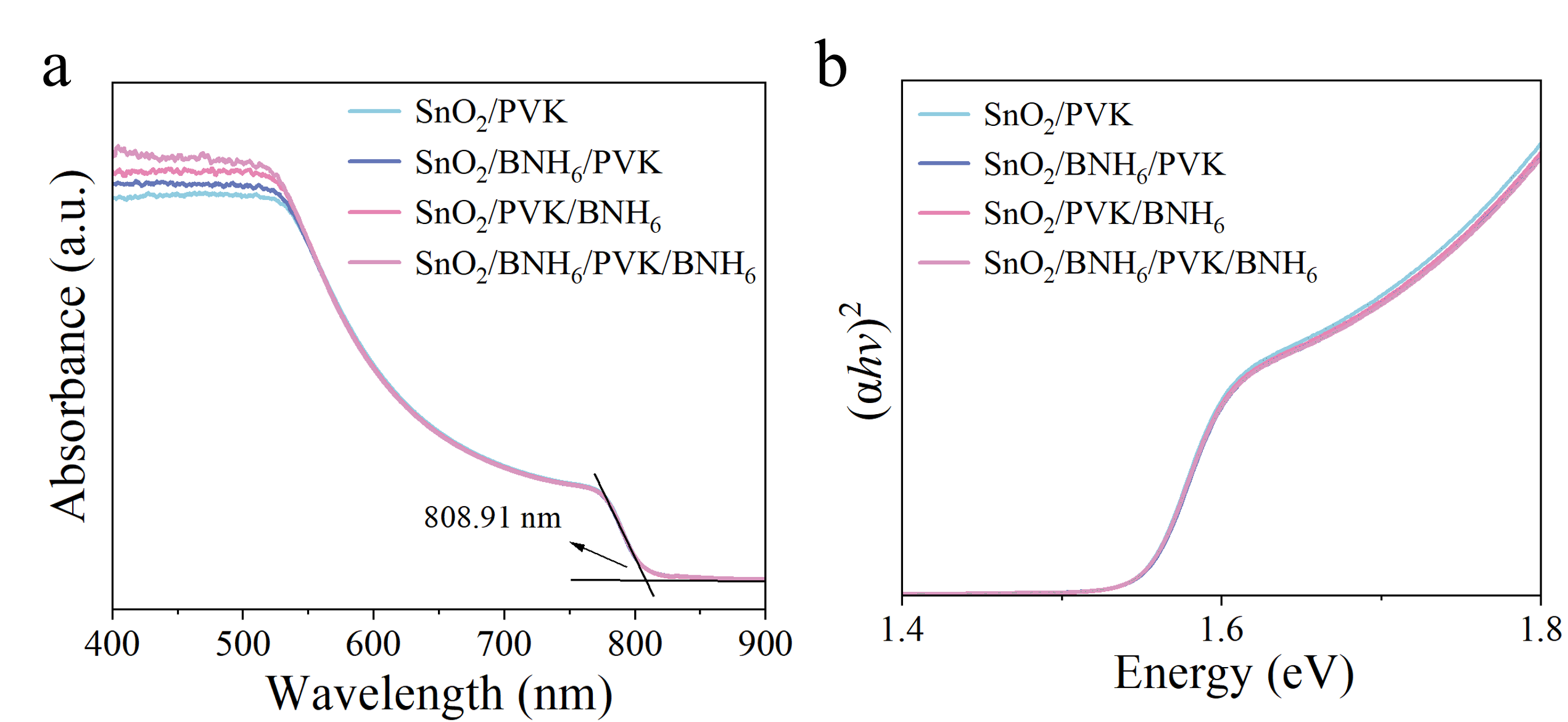


**Fig. S23** (**a**) UV-vis absorption spectra and (**b**) Tauc plots of the SnO_2_/PVK, SnO_2_/BNH_6_/PVK, SnO_2_/PVK/BNH_6_ and SnO_2_/BNH_6_/PVK/BNH_6_ films


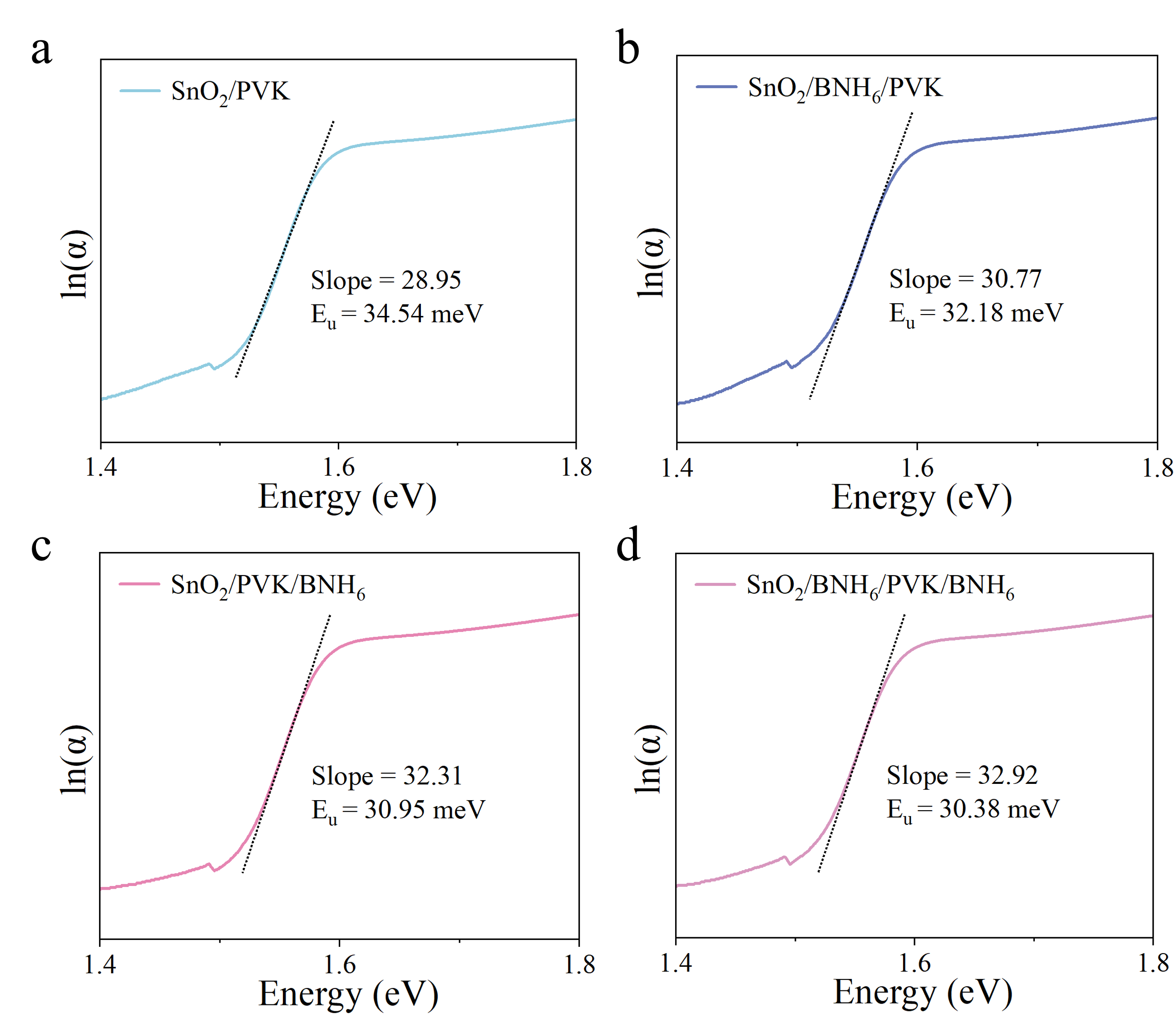


**Fig. S24** Urbach energy obtained from the (**a**) SnO_2_/PVK, (**b**) SnO_2_/BNH_6_/PVK, (**c**) SnO_2_/PVK/BNH_6_ and (**d**) SnO_2_/BNH_6_/PVK/BNH_6_ films


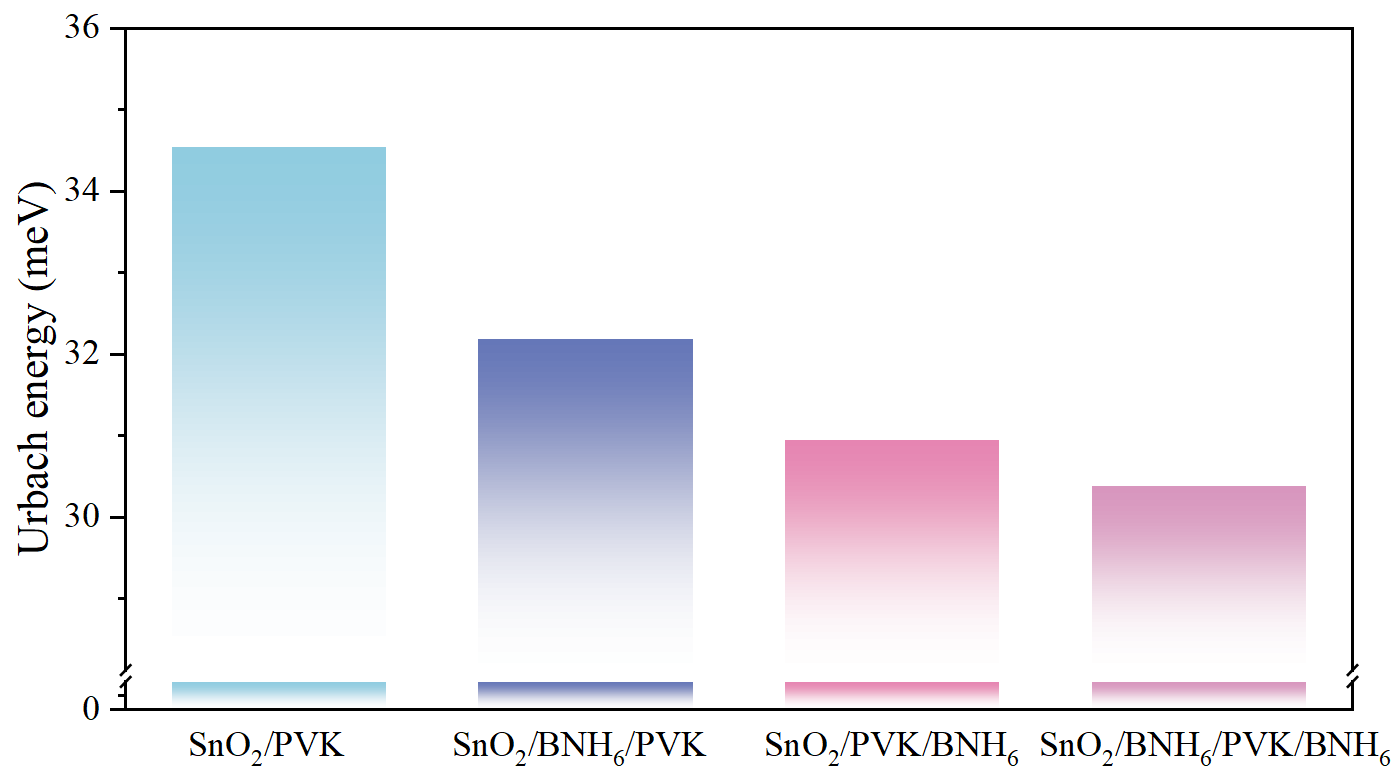


**Fig. S25** Urbach energy calculated from the SnO_2_/PVK, SnO_2_/BNH_6_/PVK, SnO_2_/PVK/BNH_6_ and SnO_2_/BNH_6_/PVK/BNH_6_ films


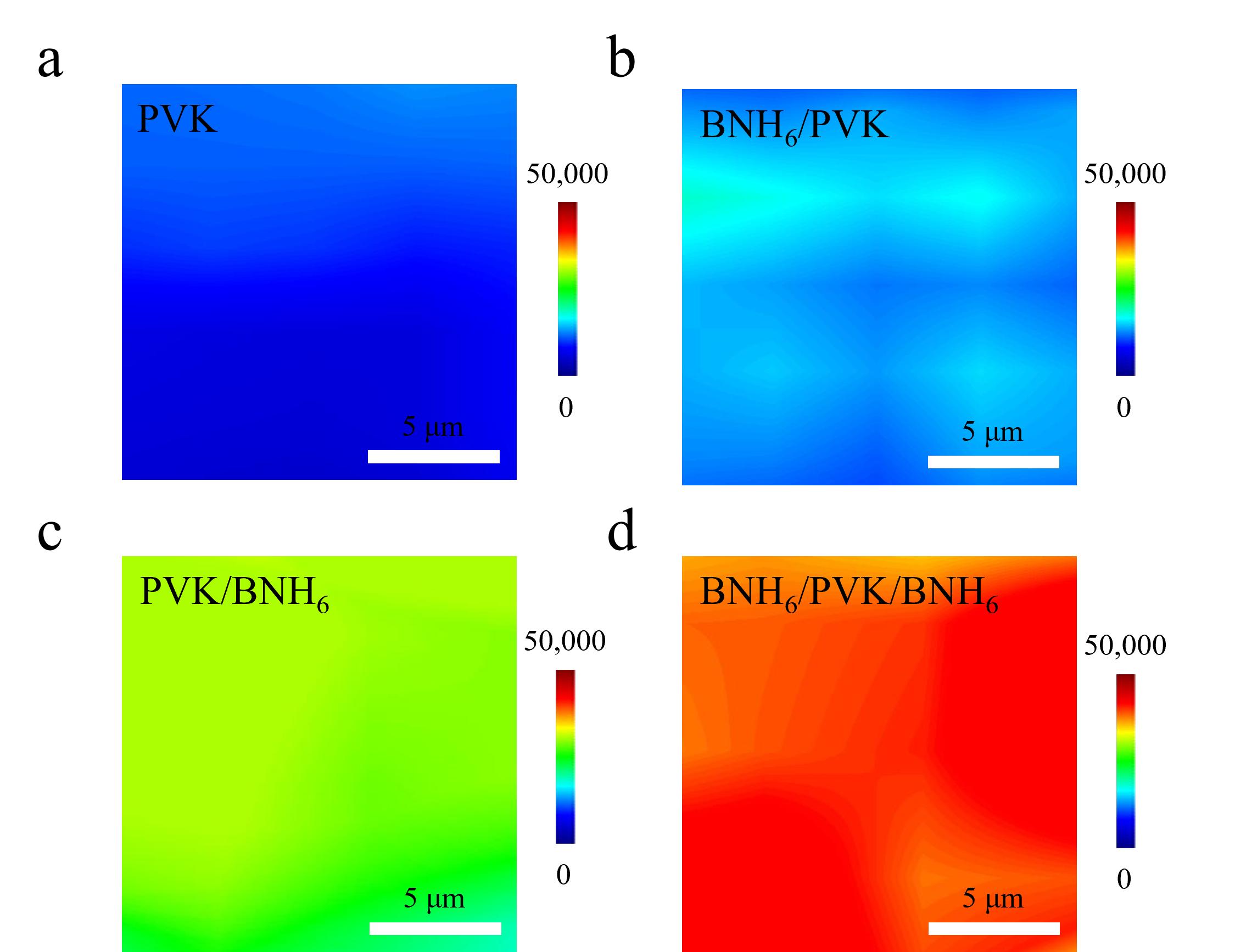


**Fig. S26** PL mapping of the (**a**) PVK, (**b**) BNH_6_/PVK, (**c**) PVK/BNH_6_ and (**d**) BNH_6_/PVK/BNH_6_ films


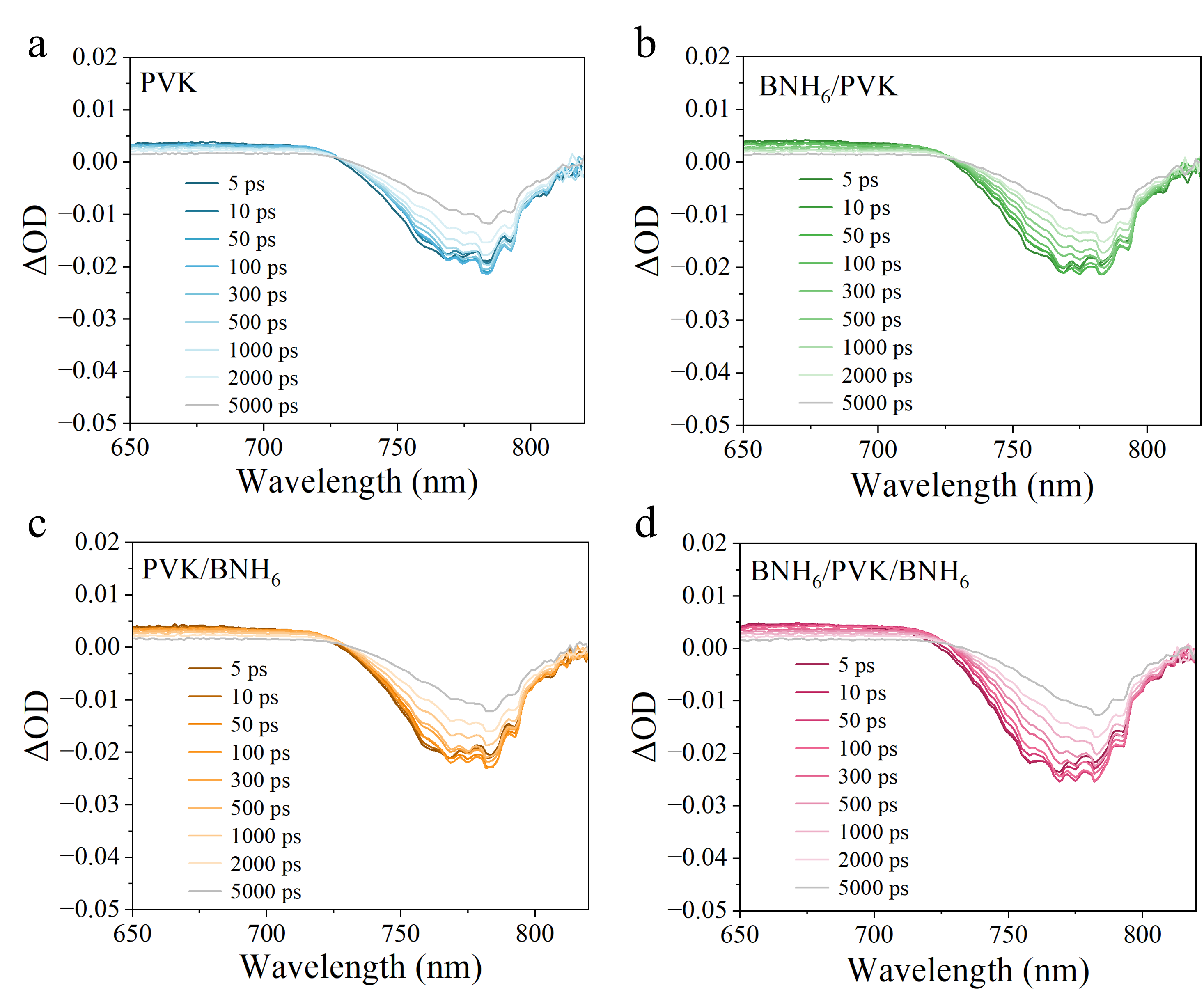


**Fig. S27** Time-resolved transient absorption of the (**a**) PVK, (**b**) BNH_6_/PVK, (**c**) PVK/BNH_6_ and (**d**) BNH_6_/PVK/BNH_6_ films at different probe delay times

**
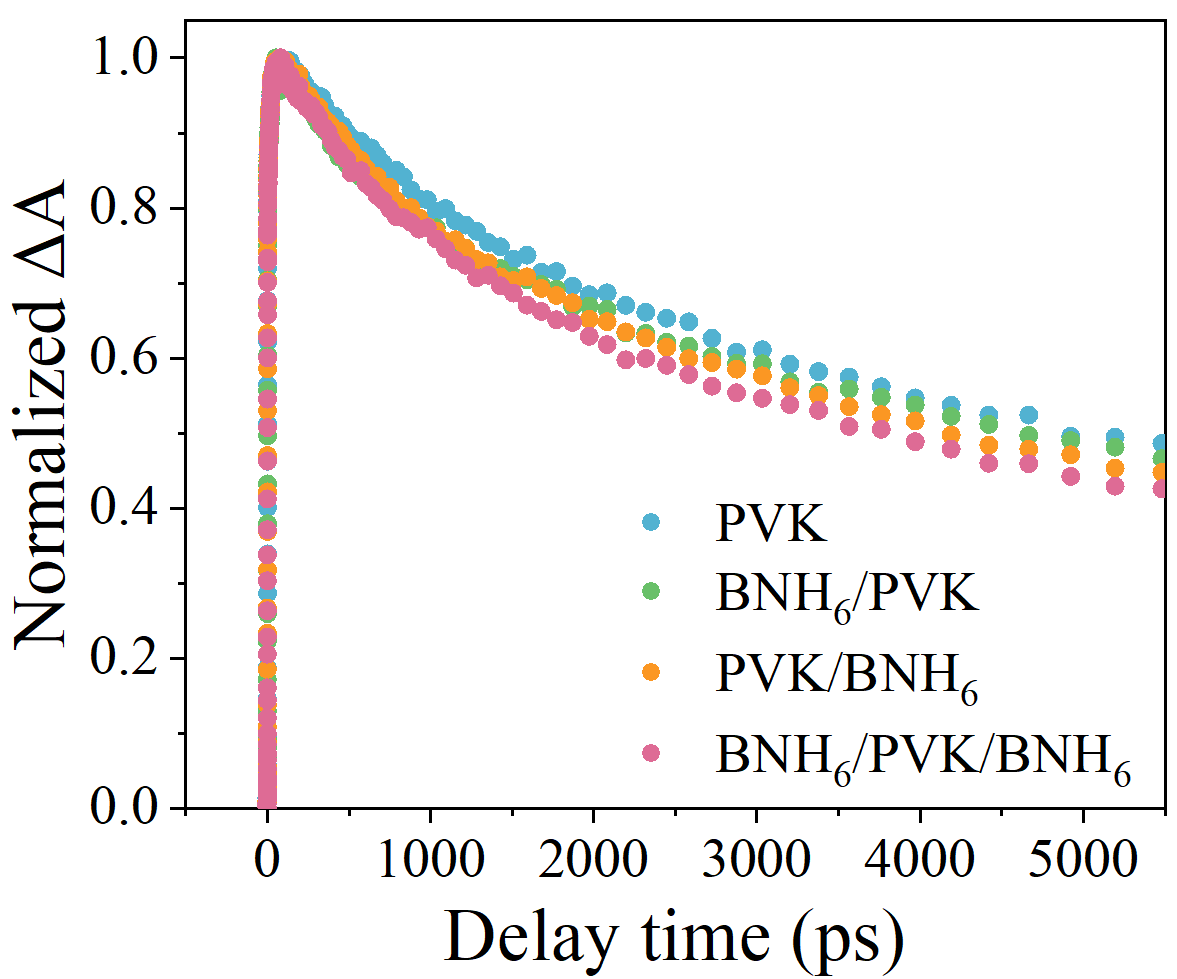
**

**Fig. S28** The decay kinetics of the corresponding GSB peaks


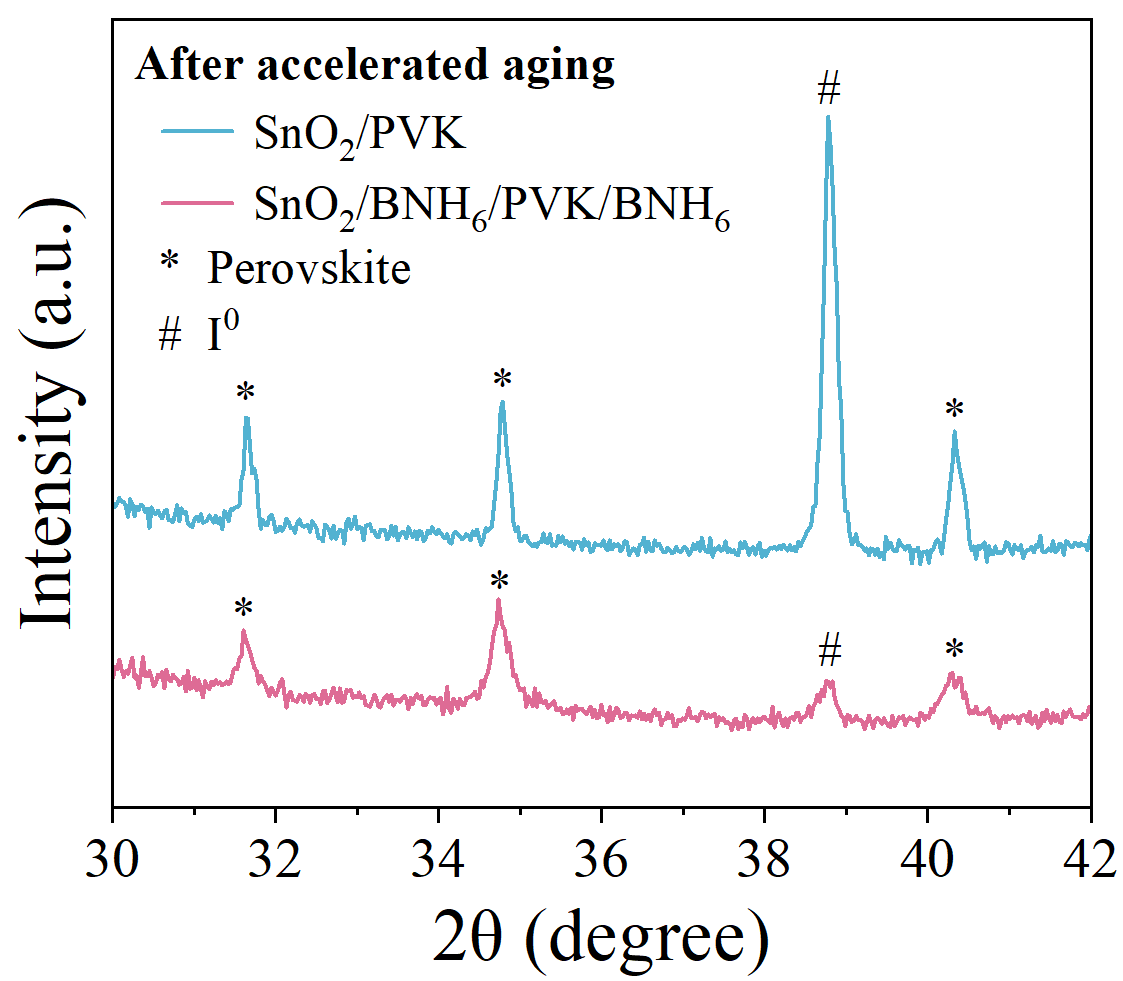


**Fig. S29** XRD spectra of the SnO_2_/PVK and SnO_2_/BNH_6_/PVK/BNH_6_ films after accelerated aging


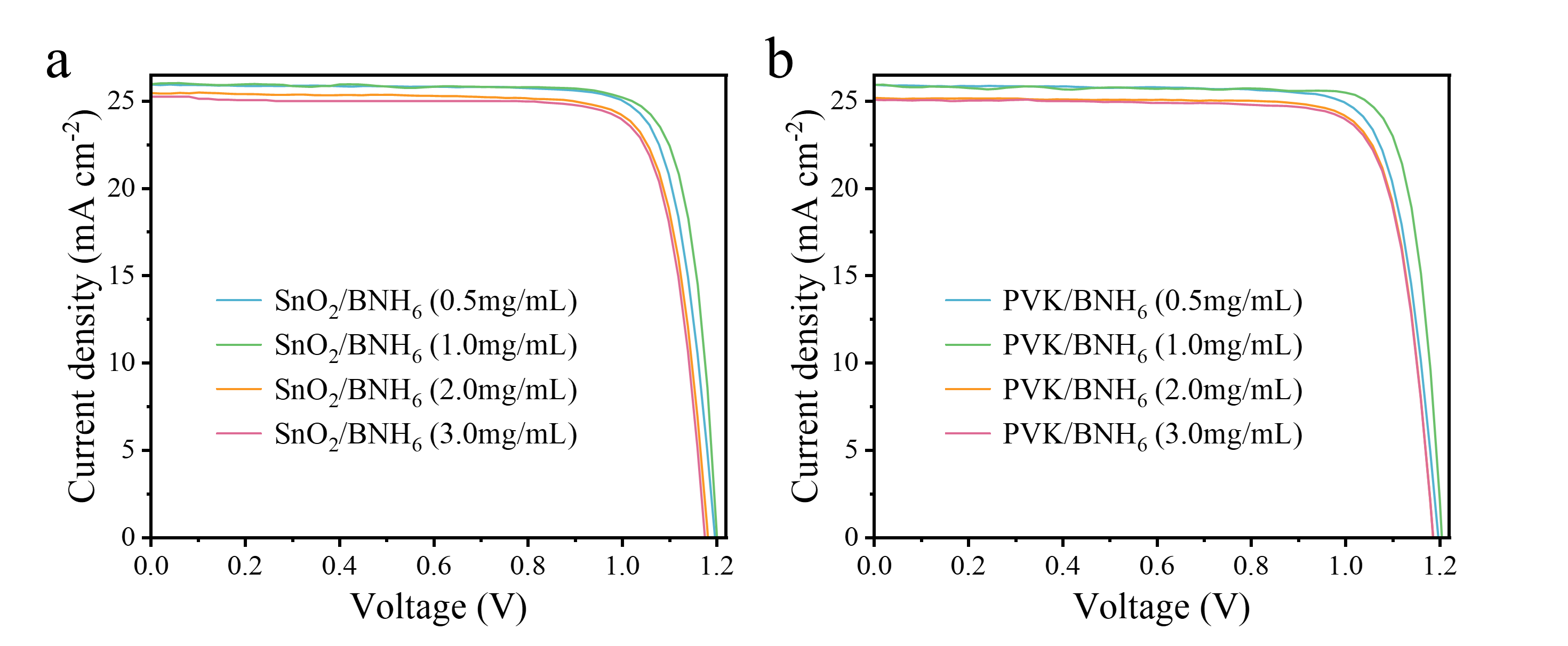


**Fig. S30** Optimal J-V curves of the PSCs measured in the reverse scan direction using different concentrations of BNH_6_. The BNH_6_ solution (0.5, 1.0, 2.0 and 3.0 mg/mL) coated at the buried and upper interface of perovskite was prepared by dissolving 0.5, 1.0, 2.0 and 3.0 mg BNH_6_ in 1 mL deionized water and 1 mL IPA, respectively. The other device preparation processes remain unchanged.


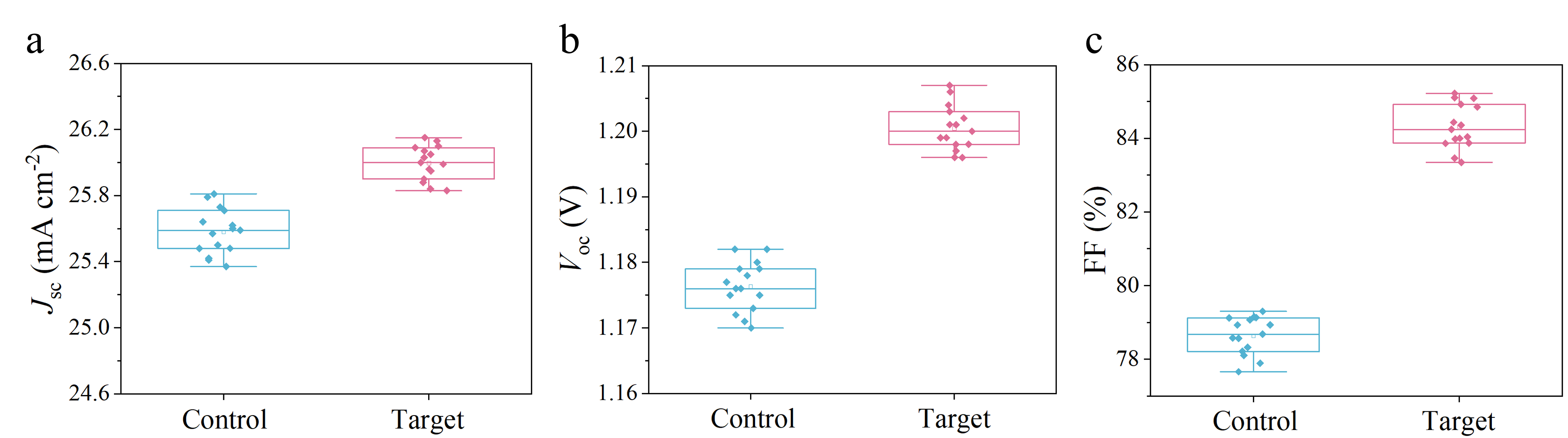


**Fig. S31** Statistical device data based on 15 devices for (**a**) *J*_sc_, (**b**) *V*_oc_ and (**c**) FF


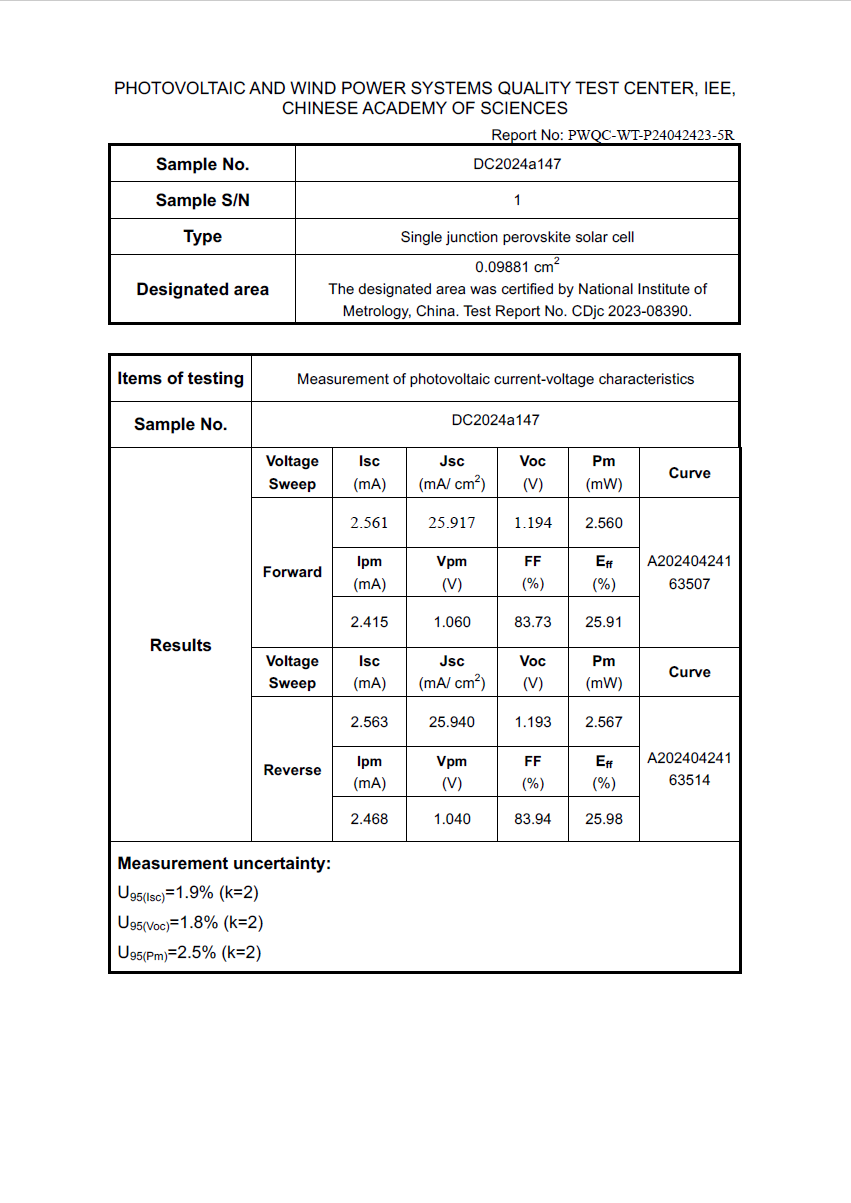

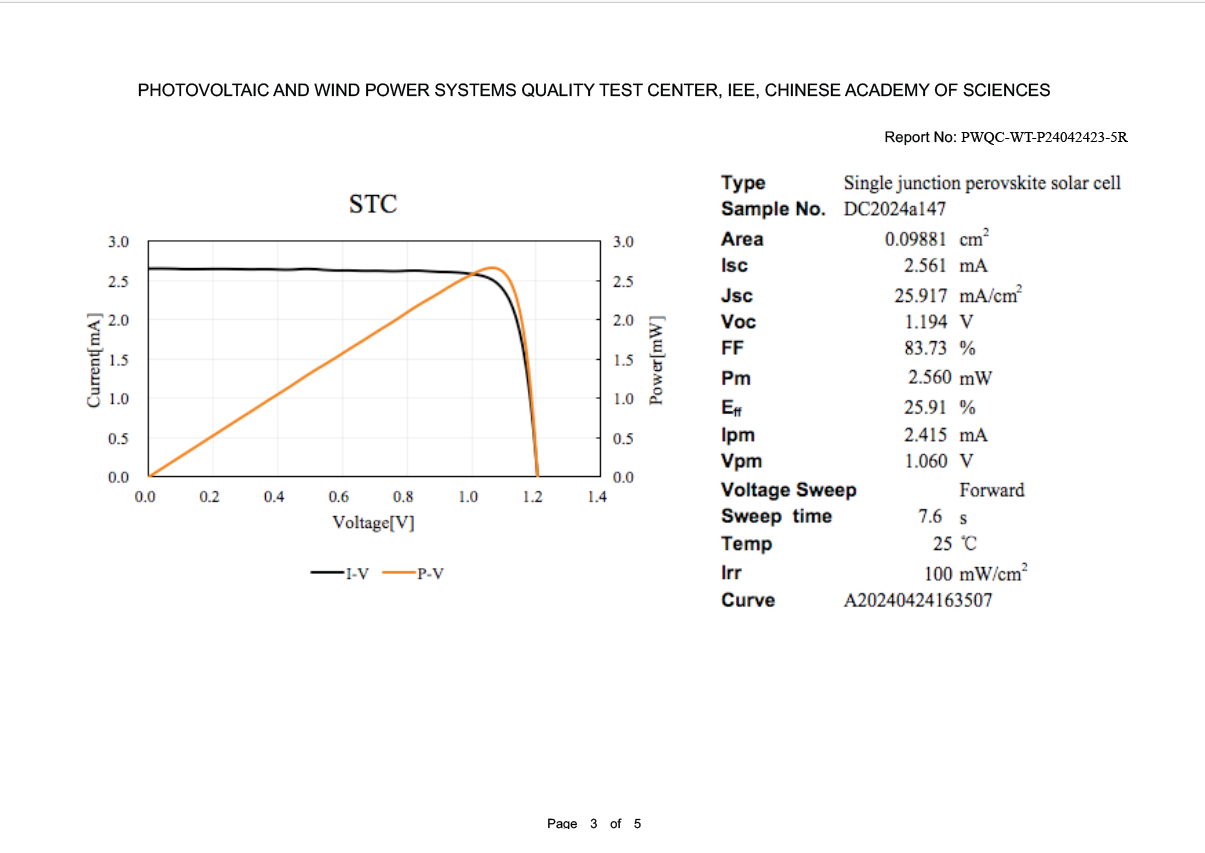


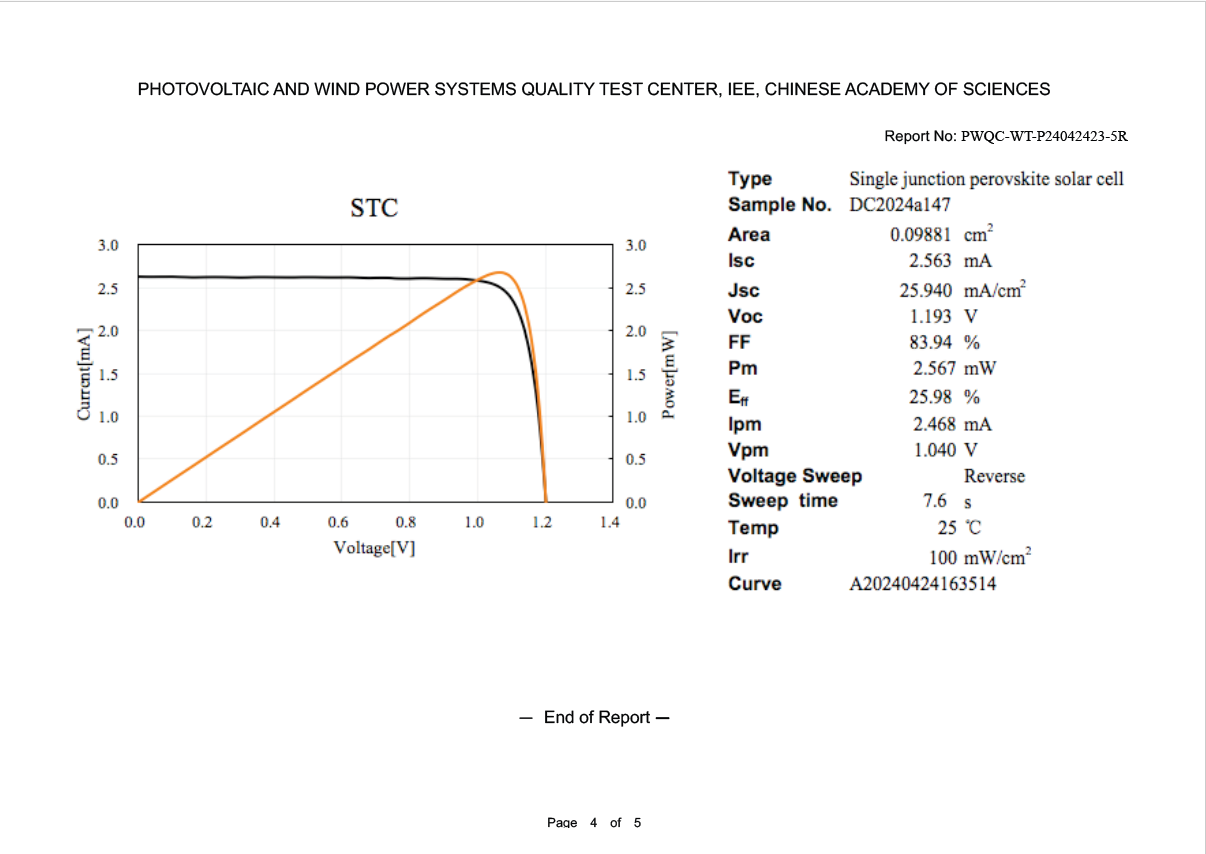


**Fig. S32** Certification of PSC from Institute of Electrical Engineering Chinese Academy of Sciences


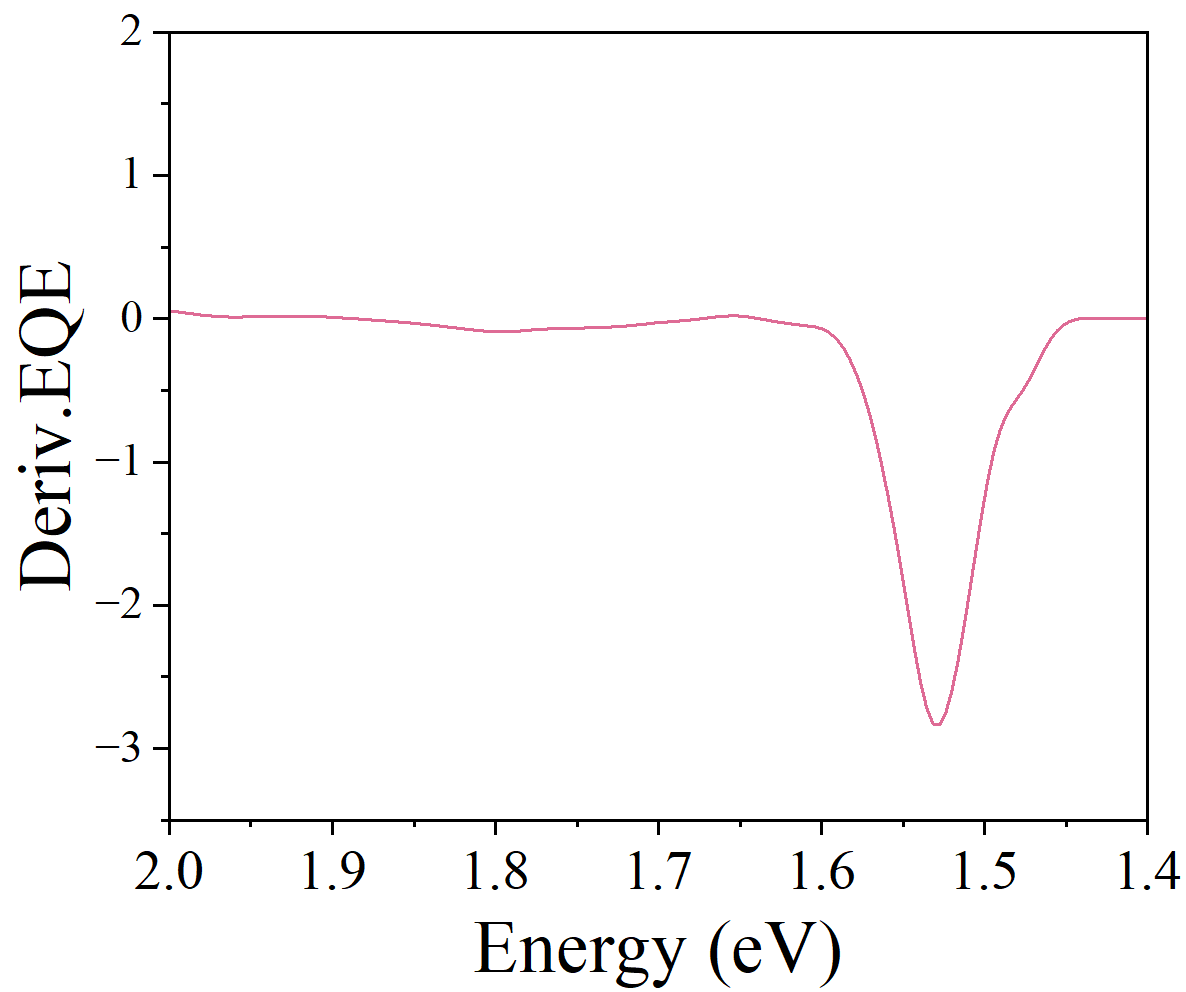


**Fig. S33** Analysis of perovskite bandgap from the EQE spectrum by taking its derivative spectrum


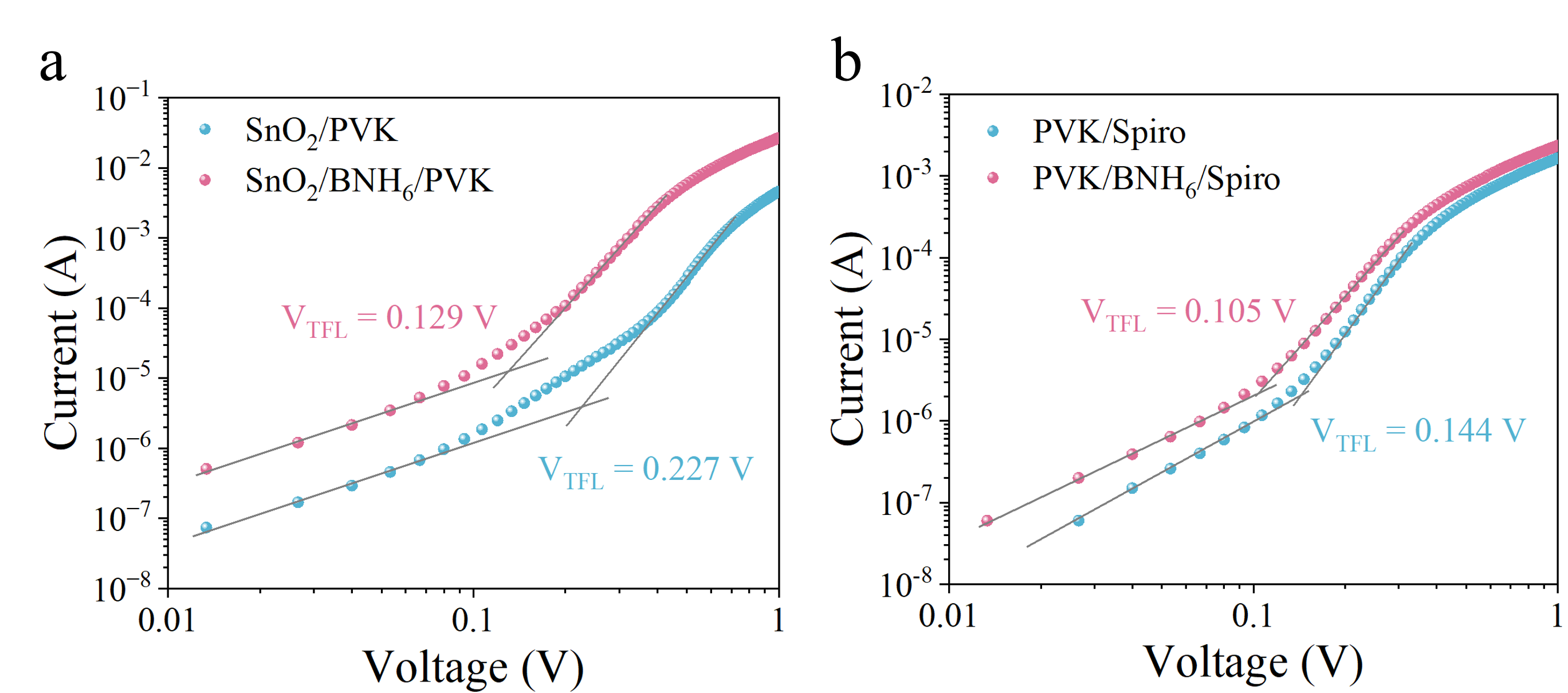


**Fig. S34** Dark I-V curves of (**a**) the electron-only devices and (**b**) the hole-only devices with and without BNH_6_


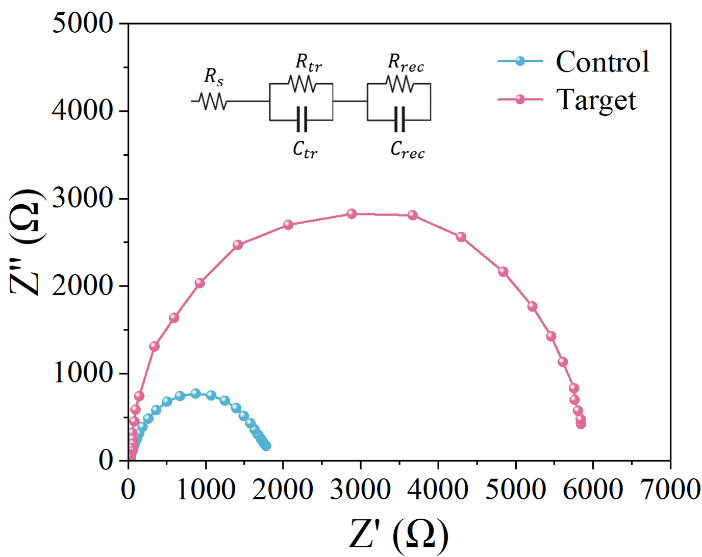


**Fig. S35** Nyquist plots of the control and target devices (Inset: Equivalent circuit model)


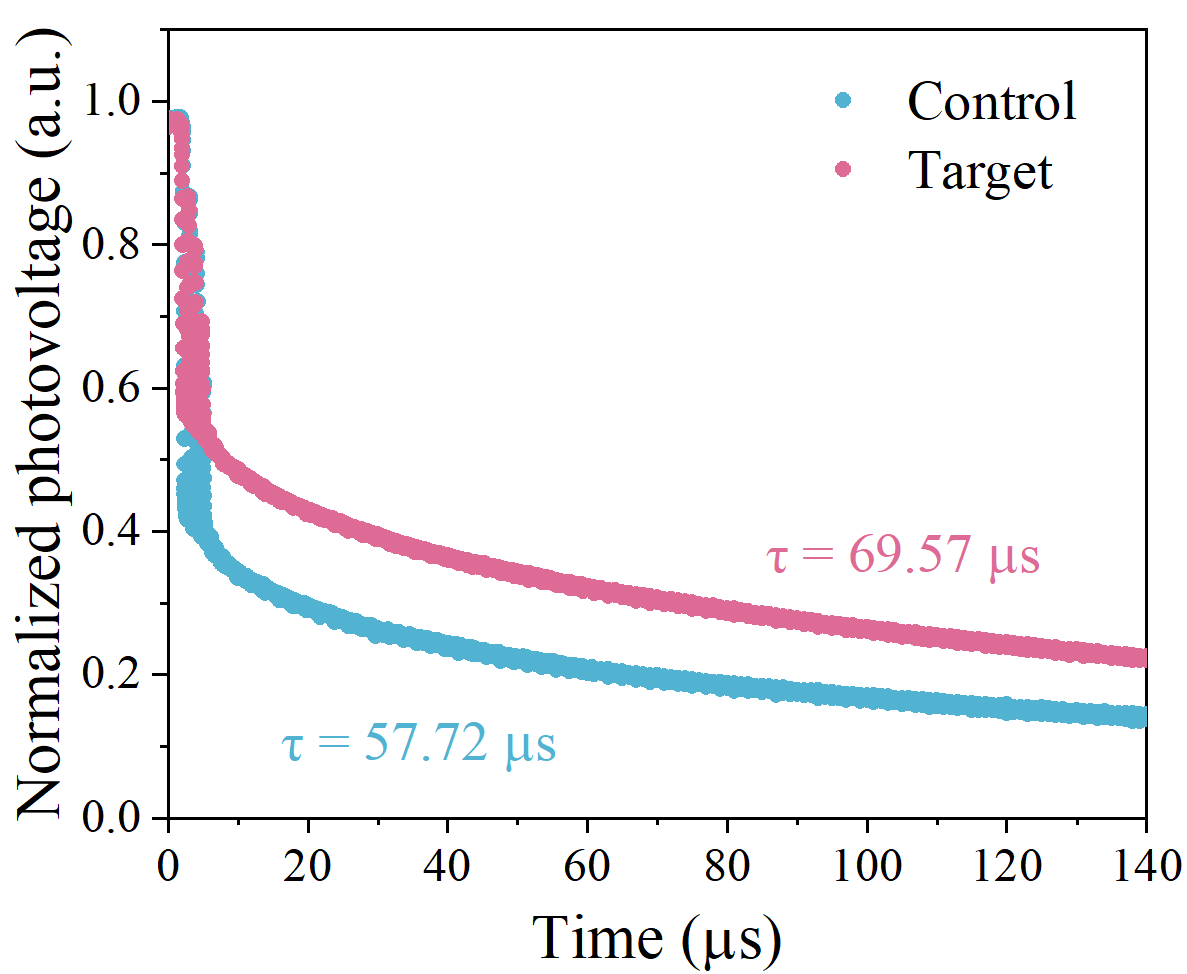


**Fig. S36** TPV of the control and target devices


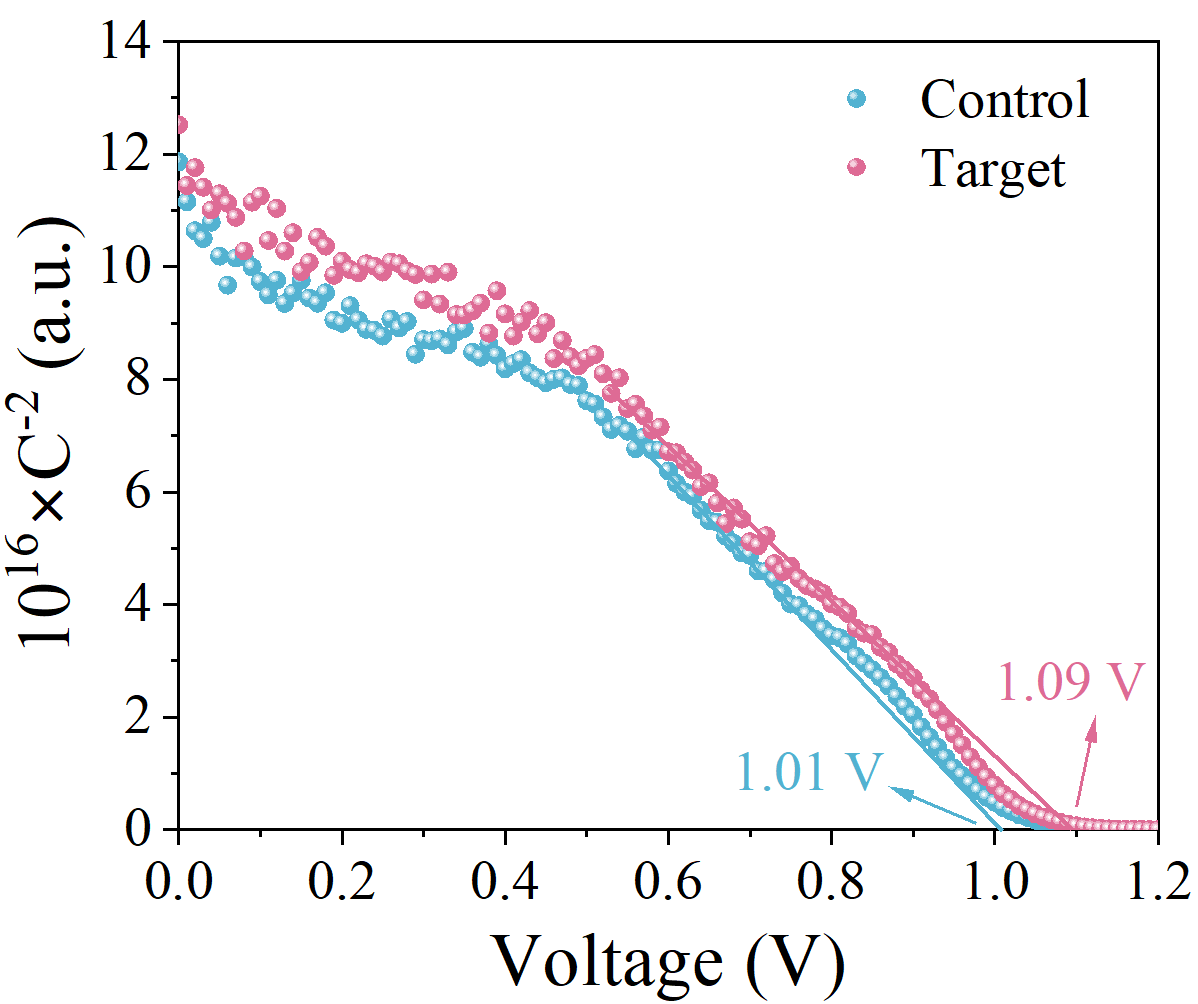


**Fig. S37** Mott-Schottky plots of the control and target devices


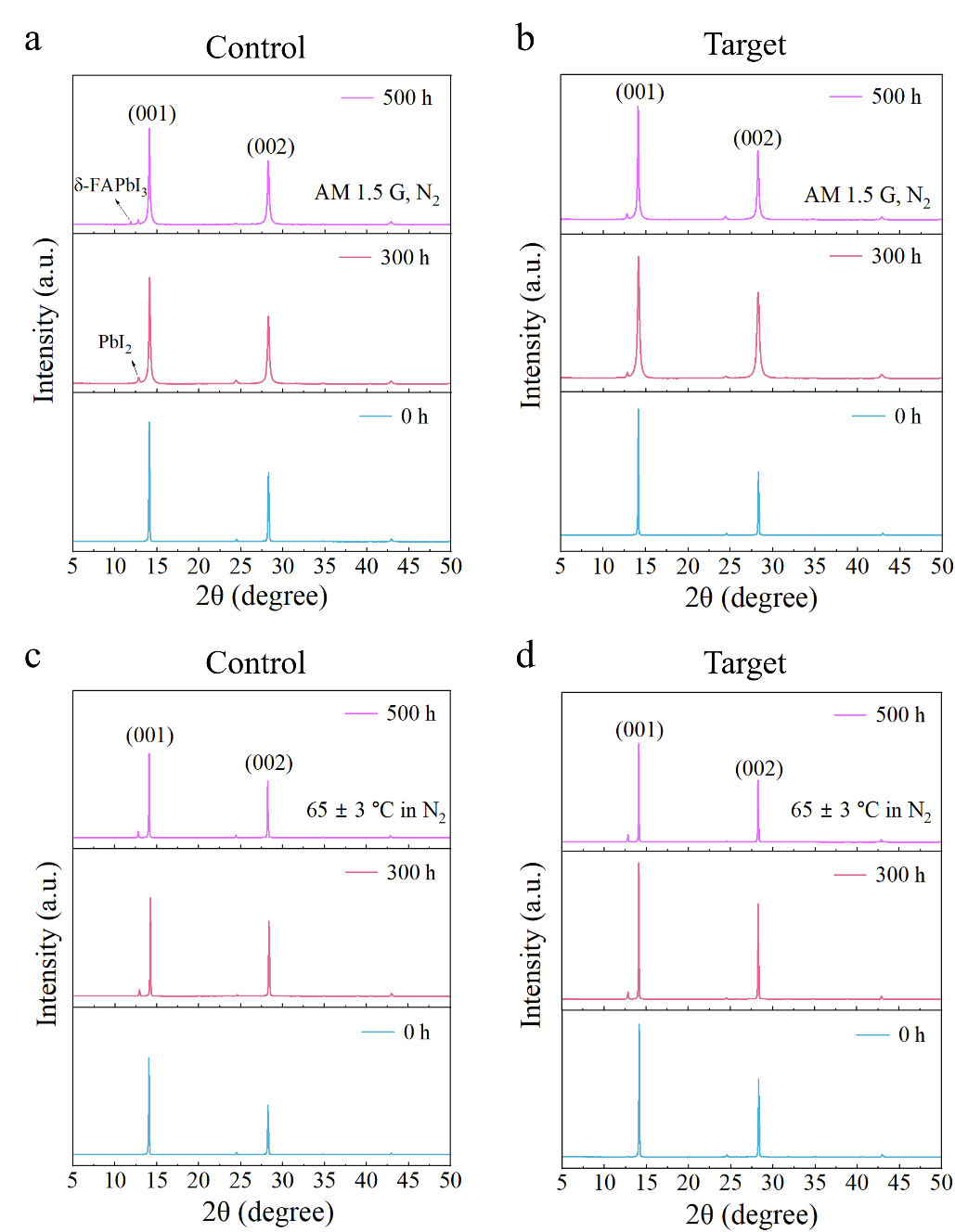


**Fig. S38** Time evolution of XRD patterns of the (**a**) control and (**b**) target device under AM 1.5 G illumination with N_2_ atmosphere, (**c**) control and (**d**) target device under 65 ± 3 °C heat with N_2_ atmosphere


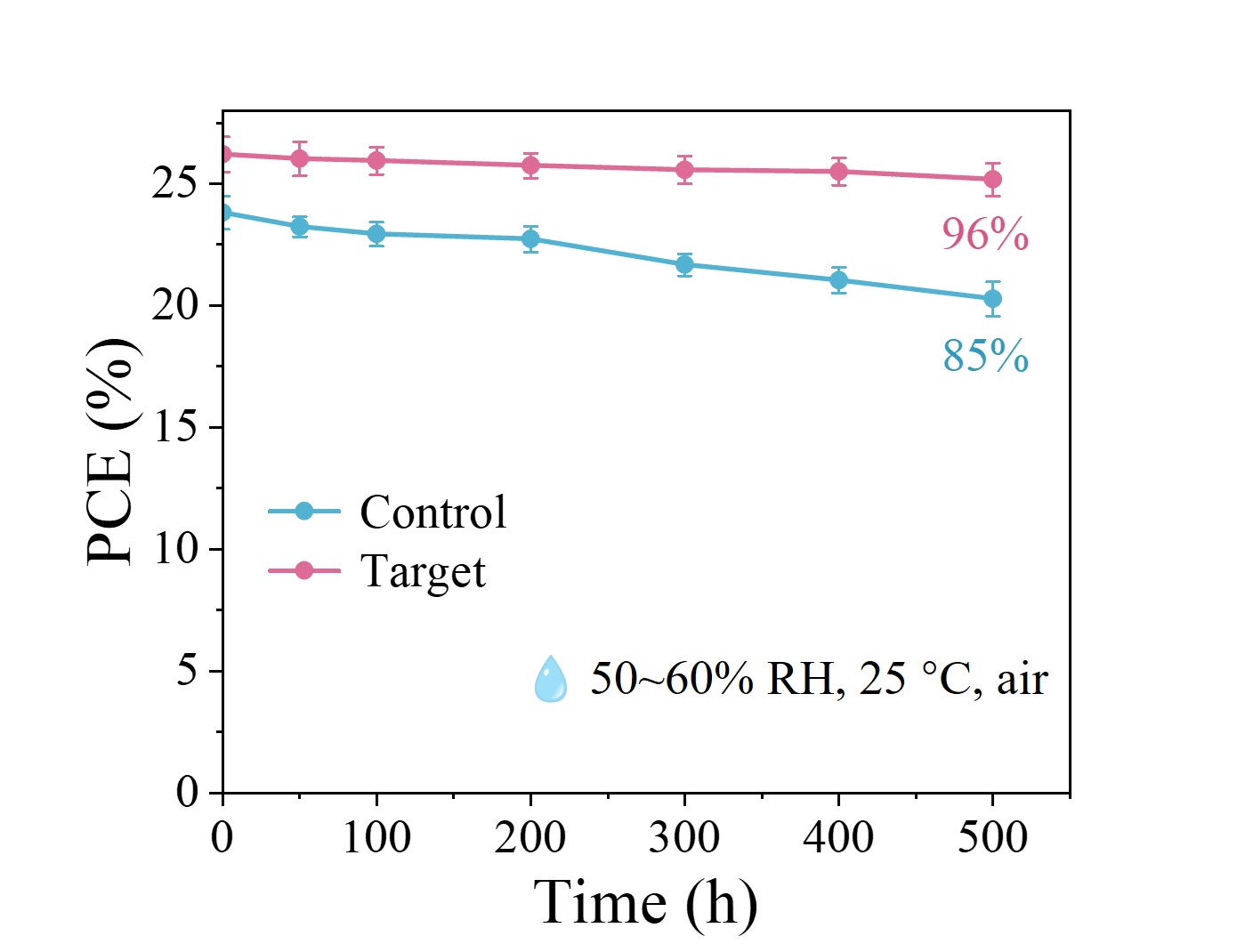


**Fig. S39** Long-term stability of the unencapsulated control and target devices stored under ambient air conditions (50~60% RH, 25 °C)


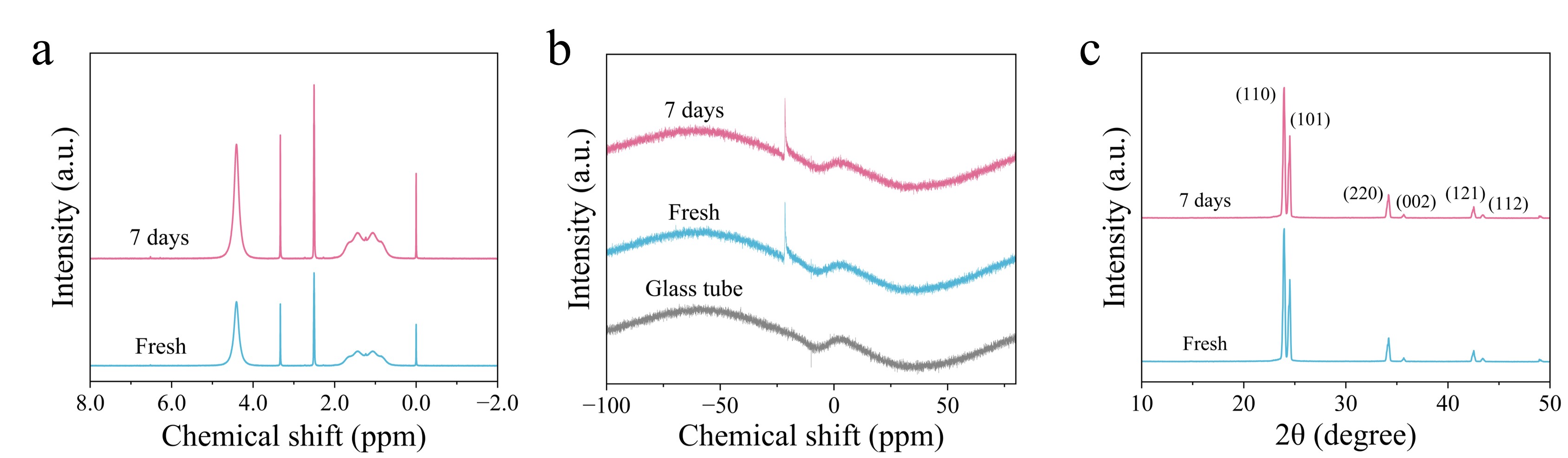


**Fig. S40** (**a**) ^1^H NMR , (**b**) ^11^B NMR and (**c**) XRD spectra of fresh BNH_6_ and BNH_6_ under accelerated conditions for 7 days. The characteristic peaks of BNH_6_ remained essentially unchanged after aging, confirming its robust chemical stability and indicating that BNH_6_ would not compromise device performance


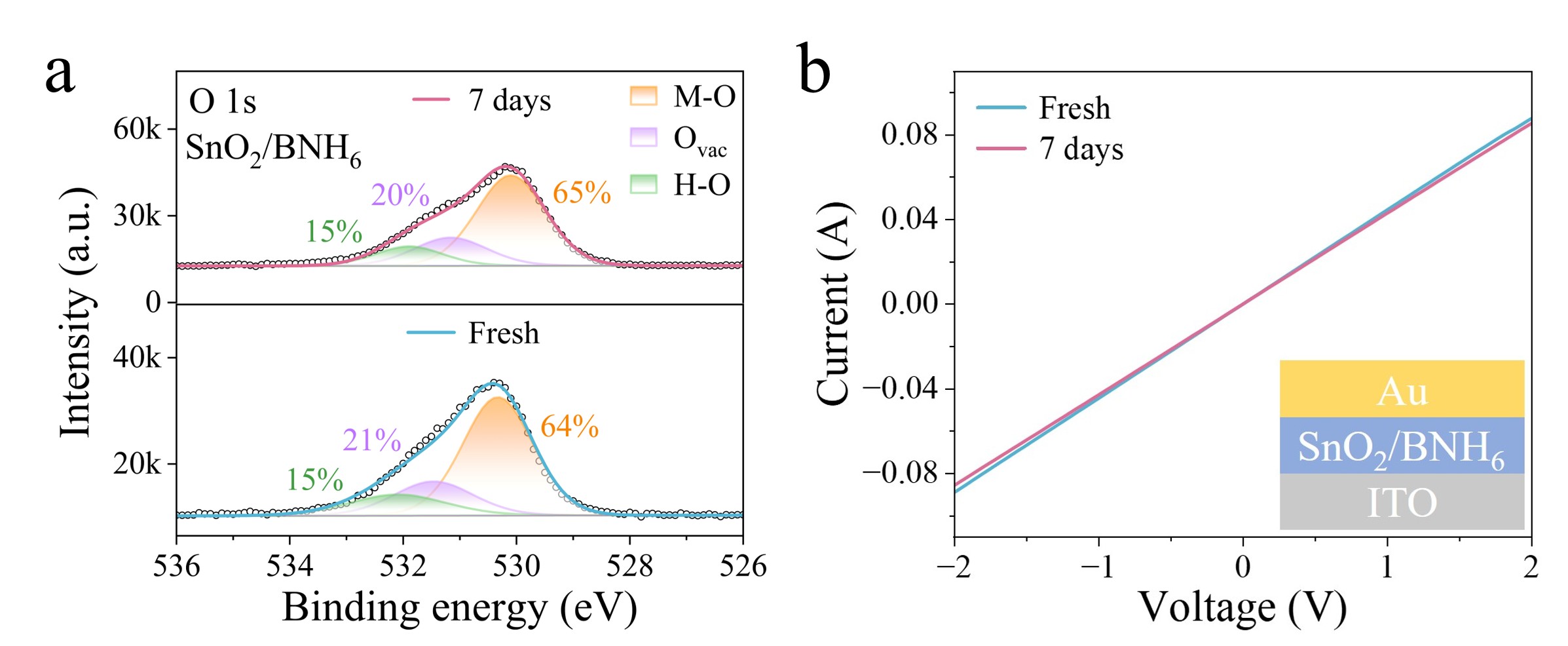


**Fig. S41** (**a**) O 1s XPS and (**b**) the electrical conductivity spectra of the SnO_2_/BNH_6_ film under accelerated conditions for 7 days. No noticeable changes in the O 1s XPS characteristic peaks were observed after aging. And the electrical conductivity measurements of BNH_6_/SnO_2_ films before and after aging showed negligible variation, further verifying the robustness of the BNH_6_/SnO_2_ interface under accelerated conditions


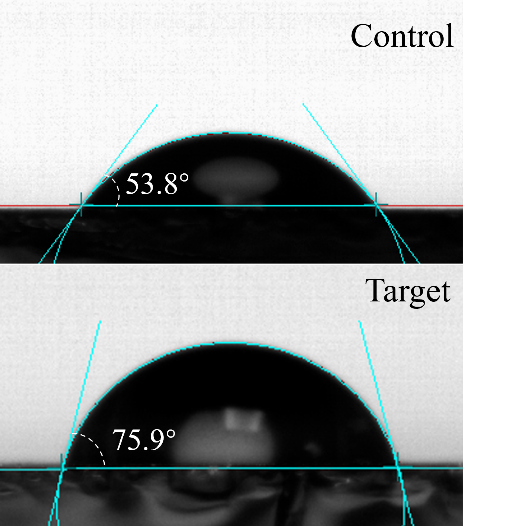


**Fig. S42** Contact angles of the control and target films

**Table S1** Summary of photovoltaic performance of the effective interface modification strategies

| Additive | Configuration | PCE  (%) | Certified PCE  (%) | Reference |
| --- | --- | --- | --- | --- |
| C_60_-SAM | p-i-n | 17.3 | na | ACS Nano **12**, 12701-12709 (2014) |
| C3-SAM | n-i-p | 15.67 | na | J. Am. Chem. Soc. **137**, 2674-2679 (2015) |
| 2D PVSK | n-i-p | 20.64 | 19.77 | Nat. Commun. **9**, 3021 (2018) |
| DPPP | n-i-p | 24.5 | na | Science **379**, 690-694 (2023) |
| HP | n-i-p | 23.68 | na | Adv. Mater. **32**, 2003990 (2020) |
| CPI | n-i-p/p-i-n | 24.3/26.0 | na | Science **384**, 878-884 (2024) |
| CBz-PAI | n-i-p | 24.7 | na | Nat. Energy **8**, 515-525 (2023) |
| 3TPYMB | p-i-n | 25.76 | na | Nat. Photon. **19**, 28-35 (2025) |
| CPMAC | p-i-n | 26.1 | na | Science **288**, 964-968 (2025) |
| TMPU/TMFS | n-i-p | 23.11 | na | Chem. Eng. J. **469**, 143790 (2023) |
| AMP/ mF-PEAI and PDI | p-i-n | 25.35 | 24.87 | Adv. Energy Mater. **14**, 2304486 (2024) |
| BNH_6_/BNH_6_ | n-i-p | 26.43 | 25.98 | This work |

Table S2 Best photovoltaic parameters of PSCs with no treatment or post treatment by oxygen plasma measured in reverse scan directions under standard AM 1.5 illumination (100 mW cm^-2^)

|  | **Samples** | ***V_oc_***  **(V)** | ***J_sc_***  **(mA cm^-2^)** | **FF**  **(%)** | **PCE**  **(%)** |
| --- | --- | --- | --- | --- | --- |
| **No treatment** | SnO_2_ | 1.171 | 25.53 | 78.08 | 23.34 |
|  | SnO_2_/BNH_6_ | 1.198 | 25.86 | 82.40 | 25.53 |
| **Post**  **treatment** | SnO_2_ | 1.176 | 25.57 | 78.56 | 23.62 |
|  | SnO_2_/BNH_6_ | 1.198 | 25.96 | 82.42 | 25.63 |

**Table S3** Parameters of the TRPL spectroscopy based on different samples

| **Samples** | **A_1_** | **τ_1_ (ns)** | **A_2_** | **τ_2_ (ns)** | **τ*_ave_* (ns)** |
| --- | --- | --- | --- | --- | --- |
| **PVK** | 158.92 | 147.28 | 748.22 | 1978.11 | 1949.61 |
| **BNH_6_/PVK** | 120.56 | 1196.47 | 750.33 | 3365.35 | 3248.15 |
| **PVK/BNH_6_** | 161.68 | 621.38 | 1108.05 | 7010.56 | 6928.98 |
| **BNH_6_/PVK/BNH_6_** | 163.98 | 1000.00 | 1119.16 | 9273.37 | 9144.68 |

Table S4 Best photovoltaic parameters of PSCs using different concentrations of BNH_6_ measured in reverse scan directions under standard AM 1.5 illumination (100 mW cm^-2^)

| **SnO_2_/BNH_6_** | **Concentration**  **(mg mL^-1^)** | ***V*_OC_**  **(V)** | ***J_SC_***  **(mA cm^-2^)** | **FF**  **(%)** | **PCE**  **(%)** |
| --- | --- | --- | --- | --- | --- |
|  | 0.5 | 1.196 | 25.93 | 81.16 | 25.18 |
|  | 1.0 | 1.198 | 25.96 | 82.42 | 25.63 |
|  | 2.0 | 1.181 | 25.47 | 80.45 | 24.20 |
|  | 3.0 | 1.174 | 25.21 | 80.80 | 23.92 |
| **PVK/BNH_6_** | 0.5 | 1.197 | 25.90 | 80.67 | 25.00 |
|  | 1.0 | 1.203 | 25.91 | 83.51 | 26.03 |
|  | 2.0 | 1.185 | 25.16 | 81.13 | 24.19 |
|  | 3.0 | 1.185 | 25.05 | 80.77 | 23.98 |

**Table S5** Photovoltaic parameters of control and target devices measured in reverse and forward scan directions under standard AM 1.5 illumination (100 mW cm^-2^)

|  | **Scan Directions** | ***V*_OC_**  **(V)** | ***J*_SC_**  **(mA cm^-2^)** | **FF**  **(%)** | **PCE**  **(%)** |
| --- | --- | --- | --- | --- | --- |
| **Control** | RS | 1.176 | 25.57 | 78.56 | 23.62 |
|  | FS | 1.171 | 25.55 | 77.26 | 23.12 |
| **Target** | RS | 1.206 | 26.05 | 84.12 | 26.43 |
|  | FS | 1.201 | 25.99 | 83.67 | 26.12 |

**Table S6** Parameters from SCLCs of the electron-only devices and of the hole-only devices

|  | **SnO_2_/PVK** | **SnO_2_/BNH_6_/PVK** | **PVK/Spiro** | **PVK/BNH_6_/Spiro** |
| --- | --- | --- | --- | --- |
| ***V*_TFL_ (V)** | 0.227 | 0.129 | 0.136 | 0.119 |
| **Trap**  **density(cm^-3^)** | 2.7910‧10^15^ | 1.5861‧10^15^ | 1.7705‧10^15^ | 1.2910‧10^15^ |

Trap densities were calculated using the V_TFL_ from SCLC measurements, and the equation $N_{traps}=2\varepsilon_{0}\varepsilon_{r}V_{TFL}/(qL^{2})$, where the ε_0_ is the vacuum permittivity, ε_r_ (47) is the relative dielectric constant of FAPbI_3_, q is the elementary charge, and L (650 nm) is the thickness of the perovskite films, respectively.

**Table S7** EIS fitting parameters of the control and target PSCs

| **Device** | ***R*_s_ (Ω)** | ***R*_rec_ (Ω)** |
| --- | --- | --- |
| **Control** | 33.28 | 1618 |
| **Target** | 29.65 | 5699 |
